# Biphenylalkoxyamine Derivatives–Histamine H_3_ Receptor Ligands with Butyrylcholinesterase Inhibitory Activity

**DOI:** 10.3390/molecules26123580

**Published:** 2021-06-11

**Authors:** Dorota Łażewska, Paula Zaręba, Justyna Godyń, Agata Doroz-Płonka, Annika Frank, David Reiner-Link, Marek Bajda, Dorota Stary, Szczepan Mogilski, Agnieszka Olejarz-Maciej, Maria Kaleta, Holger Stark, Barbara Malawska, Katarzyna Kieć-Kononowicz

**Affiliations:** 1 Department of Technology and Biotechnology of Drugs, Jagiellonian University Medical College, Medyczna Str. 9, 30-688 Kraków, Poland; a.doroz-plonka@uj.edu.pl (A.D.-P.); agnieszka.olejarz@uj.edu.pl (A.O.-M.); maria.kaleta@uj.edu.pl (M.K.); mfkonono@cyf-kr.edu.pl (K.K.-K.); 2 Department of Physicochemical Drug Analysis, Jagiellonian University Medical College, Medyczna Str. 9, 30-688 Kraków, Poland; paula.zareba@uj.edu.pl (P.Z.); justyna.godyn@uj.edu.pl (J.G.); marek.bajda@uj.edu.pl (M.B.); dorota.stary@gmail.com (D.S.); mfmalaws@cyf-kr.edu.pl (B.M.); 3 Institute of Pharmaceutical and Medicinal Chemistry, Heinrich Heine University Düsseldorf, Universitätsstr. 1, 40225 Düsseldorf, Germany; annikafrank@gmx.de (A.F.); david.reiner@sund.ku.dk (D.R.-L.); stark@hhu.de (H.S.); 4 Department of Pharmacodynamic, Faculty of Pharmacy, Jagiellonian University Medical College, Medyczna Str. 9, 30-688 Kraków, Poland; szczepan.mogilski@uj.edu.pl

**Keywords:** multi-target ligands, histamine H_3_ receptor ligands, butyrylcholinesterase inhibitors, monoamine oxidase inhibitors, Alzheimer’s disease

## Abstract

Neurodegenerative diseases, e.g., Alzheimer’s disease (AD), are a key health problem in the aging population. The lack of effective therapy and diagnostics does not help to improve this situation. It is thought that ligands influencing multiple but interconnected targets can contribute to a desired pharmacological effect in these complex illnesses. Histamine H_3_ receptors (H_3_Rs) play an important role in the brain, influencing the release of important neurotransmitters, such as acetylcholine. Compounds blocking their activity can increase the level of these neurotransmitters. Cholinesterases (acetyl- and butyrylcholinesterase) are responsible for the hydrolysis of acetylcholine and inactivation of the neurotransmitter. Increased activity of these enzymes, especially butyrylcholinesterase (BuChE), is observed in neurodegenerative diseases. Currently, cholinesterase inhibitors: donepezil, rivastigmine and galantamine are used in the symptomatic treatment of AD. Thus, compounds simultaneously blocking H_3_R and inhibiting cholinesterases could be a promising treatment for AD. Herein, we describe the BuChE inhibitory activity of H_3_R ligands. Most of these compounds show high affinity for human H_3_R (*K_i_* < 150 nM) and submicromolar inhibition of BuChE (IC_50_ < 1 µM). Among all the tested compounds, **19** (**E153,** 1-(5-([1,1′-biphenyl]-4-yloxy)pentyl)azepane) exhibited the most promising in vitro affinity for human H_3_R, with a *K*_i_ value of 33.9 nM, and for *equine* *serum* BuChE, with an IC_50_ of 590 nM. Moreover, **19** (**E153**) showed inhibitory activity towards human MAO B with an IC_50_ of 243 nM. Furthermore, in vivo studies using the Passive Avoidance Task showed that compound **19** (**E153**) effectively alleviated memory deficits caused by scopolamine. Taken together, these findings suggest that compound **19** can be a lead structure for developing new anti-AD agents.

## 1. Introduction

**Figure 1:**
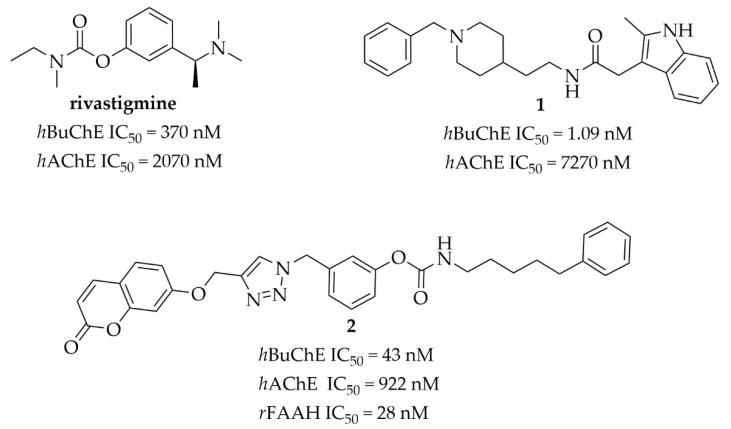
Structures and cholinesterases inhibitory activity of **rivastigmine** and selected ligands (**1** and **2**) [5]. *h*AChE–human acetylcholinesterase; *h*BuChE–human butyrylcholineserase; rFAAH–Fatty Acid Amide Hydrolase from rat brain membranes.

Acetylcholinesterase (AChE) and butyrylcholinesterase (BuChE) are enzymes catalyzing the hydrolysis of acetylcholine (ACh). BuChE is synthesized in the liver and found in most human tissues. In the human brain, BuChE is mainly expressed in regions that are important for cognition and behavior (e.g., in white matter and glia) [1]. The physiological function of BuChE is still unclear but increased activity of this enzyme has been observed in some diseases, e.g., neurodegenerative ones including Alzheimer’s disease (AD) and Parkinson’s disease (PD), uremia, obesity, hyperthyroidism and diabetes [1]. Moreover, BuChE is responsible for the hydrolysis of different esters and defense against toxic compounds, e.g., physostigmine or cocaine [1]. Furthermore, BuChE could be involved in the pathogenesis of AD as it has been found in β-amyloid plaques and neurofibrillary tangles. In vivo studies conducted with butyryl-knockout mice (5XFAD/BuChE-KO) showed diminished β-amyloid plaques deposition in these mice, especially in males [2,3]. Moreover, β-amyloid plaques are also observed in the brains of old people without AD, but BuChE accumulation is observed only in the plaques of AD. Thus, BuChE could be an appropriate biomarker for diagnosis of AD [4]. Furthermore, in advanced AD, the level of BuChE increases, while the level of AChE diminishes [1]. Thus, BuChE plays an important role in the hydrolysis of ACh in AD [5]. In the present therapy for AD, three cholinesterase inhibitors are used: donepezil, galantamine and rivastigmine. Among them, **rivastigmine** (Figure 1) is the only dual AChE-BuChE inhibitor, with IC_50_ values of 2070 nM for AChE and 370 nM for BuChE, respectively [5]. Therefore, there is a strong need to find new cholinesterase inhibitors, especially those blocking BuChE activity, which might be a better target for the therapeutic treatment of advanced AD than selective AChE inhibitors. In recent years, selective and potent BuChE inhibitors (**1**, Figure 1) as well as multitarget-directed ligands (**2**, Figure 1) have been found by using medicinal–chemistry strategies [5,6,7,8].

**Figure 2:**
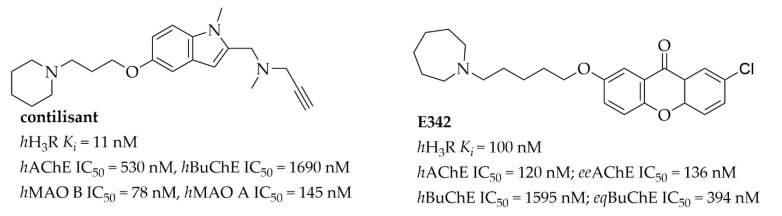
Structures and biological activity of selected multi-target histamine H_3_ receptor ligands.

Histamine H_3_ receptors (H_3_Rs) belong to the family of GPCR receptors. They are mostly present in the brain in the region connected with memory and wakefulness [9]. Since 1983, when Arrang et al. [10] first characterized this receptor in rats, many potent and selective H_3_R ligands, mostly inhibiting their activity, have been described—for review of antagonists/inverse agonists see: [11,12]. The pharmacology of H_3_R is complicated, as many splices have been described with different functions, and furthermore receptors show constitutive activity and can be activated spontaneously without the presence of an agonist [9]. Initially, H_3_R ligands were described as single target compounds, but recently, as the general tendency in medicinal chemistry has changed [13], multi-targeting ligands have been reported [14]. Especially interesting are compounds designed with potential utility in the treatment of AD, such as **contilisant** or compound **E342** (Figure 2) [15,16].

**Figure 3:**
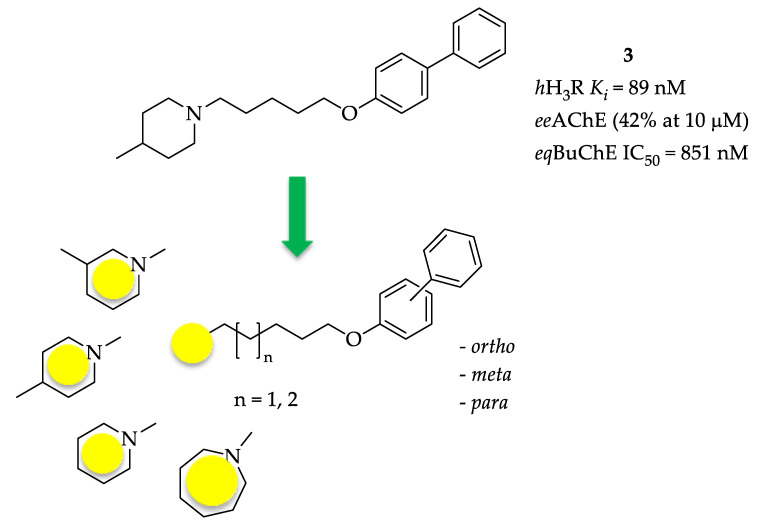
Structures of investigated compounds.

This work is a continuation of our previous studies concerning multi-target H_3_R compounds [16,17]. Preliminary docking to the active site of AChE of selected structures from our database of histamine H_3_R ligands showed high scoring values (46.13–7.57) by biphenyloxyamine derivatives 3–5 (Table 1) [17]. These compounds exhibited a moderate level of affinity for human H_3_R (*h*H_3_R) in the nanomolar concentration range (*K*_i_: 89–266 nM). Research conducted in vitro confirmed AChE inhibitory activity with IC_50_ values in the micromolar range for the most potent compounds and showed inhibitory activity of these compounds towards BuChE, with IC_50_ in the submicromolar range [17]. However, compound **3** (Figure 3) proved to be especially interesting—a potent human H_3_R ligand (*K_i_* = 89 nM) and a moderate BuChE inhibitor (IC_50_ = 851 nM) with very weak inhibition of AChE (42% at 10 µM).

Encouraged by these results, we decided to develop this series of multifunctional ligands. First, eight new molecules were synthesized in order to extend knowledge on the structure–activity relationship in this series of biphenyl derivatives. They differ in the length of the alkyl linker, the amine fragments (piperidine, 3- or 4-piperidine, azepane) and the type of biphenyl linkage (*ortho, meta, para*) (Figure 3). For the newly synthesized compounds, affinity for human H_3_R was determined as well as inhibitory activity towards AChE from the *electric eel* (*electrophorus electricus, ee*AChE) and BuChE from *equine serum* (*eq*BuChE). Moreover, all compounds were screened for human monoamine oxidase (*h*MAO) B inhibitory activity, as research showed that the MAO B level is increased in AD neurons and overexpression of this enzyme enhances β-amyloid production via γ-secretase [20]. Furthermore, molecular modelling studies were carried out to quantify the protein-ligand interactions with cholinesterases, and the most promising ligand was selected for in vivo studies to evaluate its ability to reverse the cognitive impairments caused by scopolamine in the passive avoidance test.

## 2. Results and Discussion

### 2.1. Chemistry

**Scheme 1:**
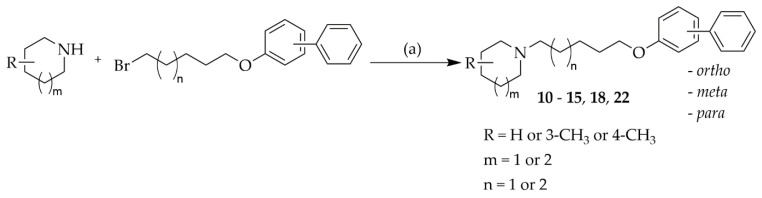
Synthesis of target compounds **10–15**, **18,** and **22** Reagents and conditions: (a) K_2_CO_3_, KI, ethanol:water (21:4, *v*/*v*), reflux 15–24 h.

The synthesis and chemical purity of 12 compounds was described previously [18]. New compounds (**10–15**, **18,** and **22**) were obtained in a similar way. The synthetic pathway is shown in Scheme 1. Briefly, suitable amines underwent *N-*alkylation with appropriate biphenyloxyalkyl bromides in a mixture of ethanol-water (21:4) in the presence of potassium carbonate. The identity and purity of final compounds were confirmed by LC-MS, elemental analysis and spectroscopic data (^1^H-NMR, ^13^C-NMR).

### 2.2. Biological Evaluation

#### 2.2.1. Affinity for the Histamine H_3_ Receptor

Affinity for the human histamine H_3_ receptor (*h*H_3_R) was evaluated in the binding assay as described previously [18]. *h*H_3_R was stably expressed in HEK293 cells and [^3^H]*N^α^*-methylhistamine was used as a radioligand (*K*_D_ = 3.08 nM [21]). All compounds showed binding affinity in a nanomolar concentration range (*K*_i_: 19-561 nM, Table 1) [18]. The in vitro potency was influenced by: a) the amine moiety (piperidine, 3- or 4-piperidine, azepane), b) the length of the carbon chain (five or six carbons), and c) the position of the introduced second phenyl (*ortho*, *meta* or *para*). Generally, in the *ortho*-substituted series (**10**, **15**, **18** and **22**), the introduction of a second phenyl into the 2-position of the phenyl ring strongly decreased the affinity (except compound **15**). Moreover, the lowest potency was shown by 4-methylpiperidine derivatives (**3**,**4**,**16–18**), whereas the five-carbon linker was the most profitable for affinity.

#### 2.2.2. Acetyl- and Butyrylcholinesterase Inhibition

The inhibitory activities of the tested compounds against cholinesterases were evaluated by Ellman’s method [22]. AChE was obtained from *electric eel* and BuChE from *equine serum*. Donepezil and rivastigmine were used as reference compounds. First, all ligands were screened at a concentration of 10 μM and then, for compounds which showed more than 50% inhibition, IC_50_ values were obtained (Table 1**)**. Despite that, five compounds (**4**, **5**, **13**, **14** and **21**) displayed sufficiently strong AChE inhibition (>50%), the obtained IC_50_ values showed only weak activity of them, with IC_50_ > 2.70 μM. All these compounds possess the hexyl linker with *para* or *meta* substituted biphenyl rings. In contrast, the tested compounds showed inhibitory activity towards BuChE in the submicromolar range with IC_50_ values below 2.20 μM. It is difficult to find any correlation between the length of the carbon linker (five or six atoms) and the observed inhibitory activity towards BuChE. However, it is noticeable that all azepane derivatives showed inhibitory activity below 1 μM, with the most potent compounds being: **5** (IC_5*0*_ = 0.60 μM), **19** (IC_50_ = 0.59 μM) and **20** (IC_50_ = 0.47 μM), which were about three-times more active than donepezil (IC_50_ = 1.83 μM) and about four-times more active than rivastigmine (IC_50_ = 2.2 μM). Furthermore, among the strongest BuChE inhibitors, the most promising was **19** with a high affinity also for *h*H_3_R (*K*_i_ = 34 nM). The other active compounds **5** and **20** showed only moderate affinity for *h*H_3_R, with *K*_i_ of 151 nM and 115 nM, respectively.

**Figure 4:**
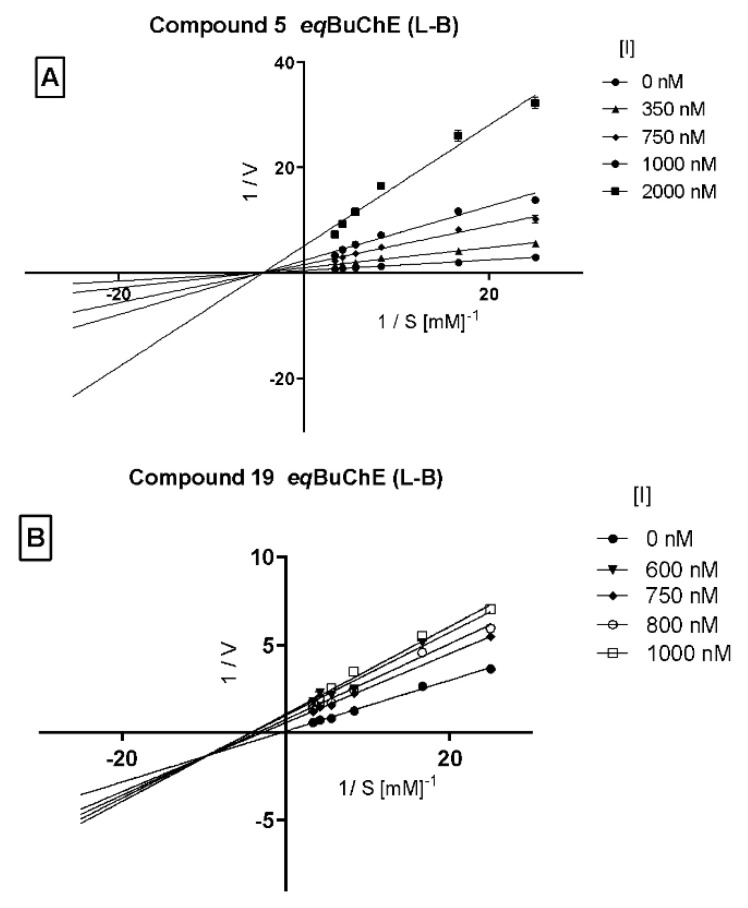
Lineweaver–Burk plot illustrating non-competitive type of *eq*BuChE inhibition by compound **5** panel (**A**) and mixed type of enzyme inhibition by compound **19** panel (**B**). S = substrate (BTC; butrylthiocholine); V = initial velocity rate; I = inhibitor concentration.

Moreover, a kinetic analysis was performed in order to investigate the mechanism of action of the two selected potent BuChE inhibitors (**5** and **19**). A double-reciprocal Lineweaver–Burk plot for compound **5** (Figure 4A) was linear and intersected at the *X*-axis (*K*_m_ remained unaffected while *V*_max_ decreased with increasing concentration of the tested inhibitor) indicating non-competitive inhibition of this compound. On the other hand, a Lineweaver–Burk plot for compound **19** (Figure 4B) shows the intersection below the *X*-axis, indicating a mixed type of enzyme inhibition.

**Figure 5:**
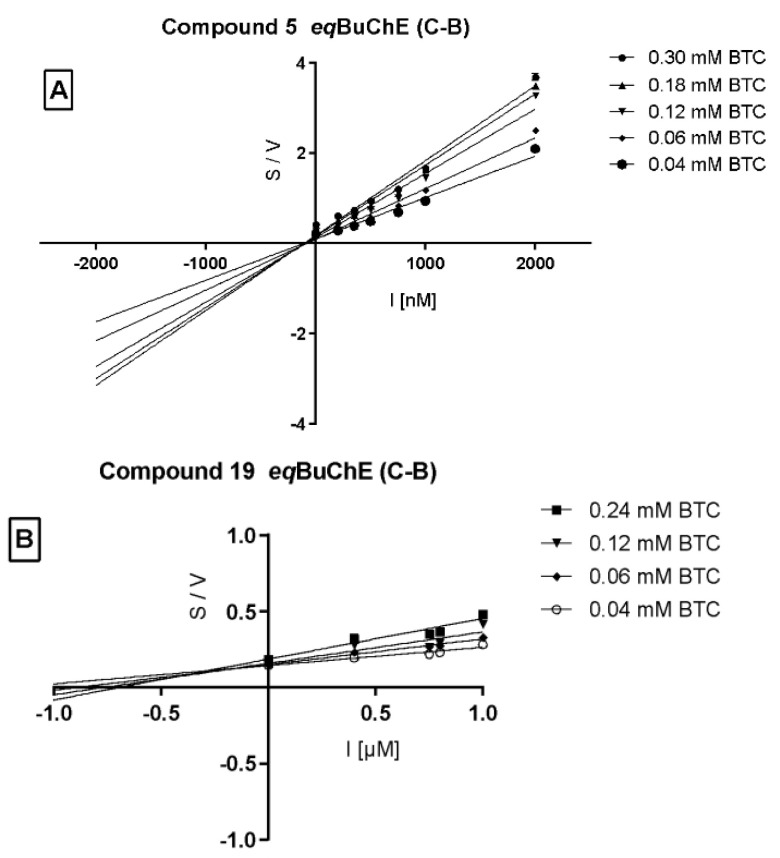
Cornish–Bowden plot illustrating non-competitive type of *eq*BuChE inhibition by compound **5** panel (**A**) and mixed type of enzyme inhibition by compound **19** panel (**B**). S = butyrylthiocholine; V = initial velocity rate, I = inhibitor concentration.

**Figure 6:**
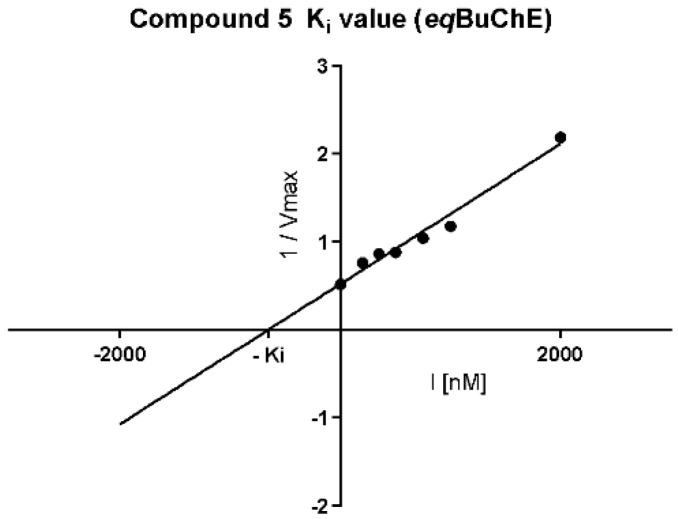
Replot from data from Lineweaver–Burk plots for determination of *K*_i_ value for non-competitive *eq*BuChE inhibitor **5**. Determined *K*_i_ = 651.5 nM, p*K*_i_ = 6.19. I = inhibitor concentration, V_max_ = maximal velocity rate.

Cornish–Bowden (Figure 5A,B) plots confirmed non-competitive *eq*BuChE inhibition by **5** and mixed mode of enzyme inhibition by **19**. Moreover, the dissociation constant (*K*_i_), describing the binding affinity between the inhibitor and the enzyme, was determined. The obtained *K*_i_ value for the non-competitive inhibitor **5** (Figure 6) was 651.5 nM.

#### 2.2.3. Human MAO B Inhibition

**Figure 7:**
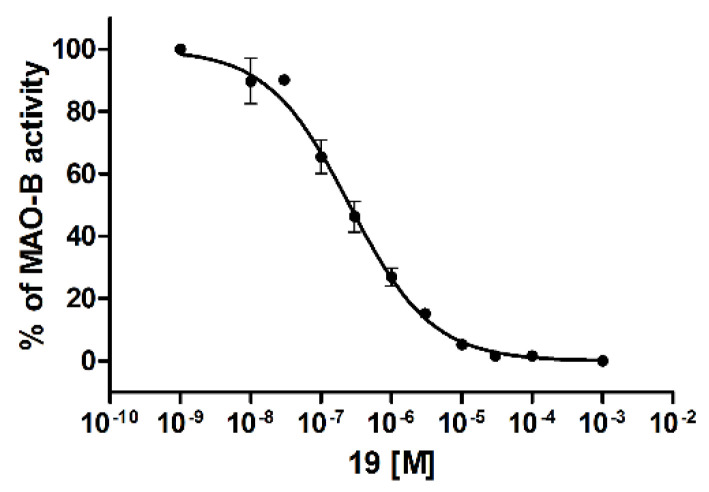
Human monoamine oxidase B IC_50_ curve of compound **19**. Data are given as mean ± standard error of the mean of one representative experiment performed in duplicate.

Inhibition of human MAO B (*h*MAO B) was evaluated using a fluorometric Monoamine Oxidase assay [23] as we described previously [24]. Firstly, the compounds were tested at a concentration of 1 µM and if the percentage of *h*MAO B inhibition was higher than 50%, an IC_50_ value was determined. In total, 19 out of 20 biphenyls showed very weak inhibition of *h*MAO B with <50% inhibition. (Table 1). Interestingly, **19** was the only compound that displayed higher inhibition and inhibited *h*MAO B with an IC_50_ of 243 nM (Figure 7).

### 2.3. Molecular Docking Studies

#### 2.3.1. Molecular Docking to Acetylcholinesterase

**Figure 8:**
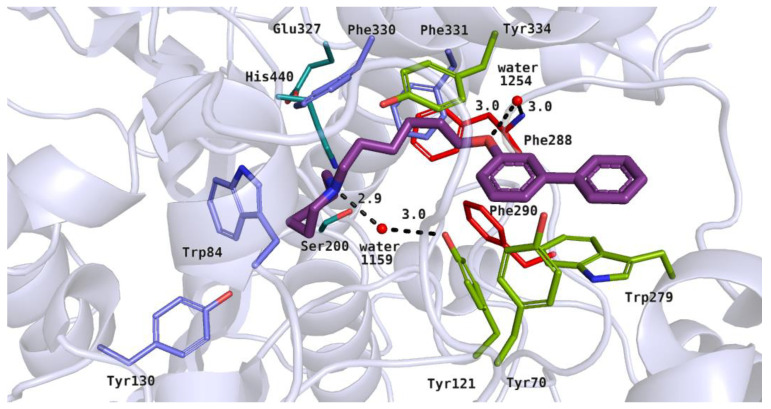
Binding mode of compound **21** within the acetylcholinesterase active site. Residues colored in teal mean catalytic triad, blue—anionic site, green—peripheral anionic site, red—acyl pocket.

Docking studies on 20 biphenyl derivatives allowed us to describe their binding mode in the active site of acetylcholinesterase. All the active compounds (**4**, **5**, **13**, **14** and **21**) showed poses similar to the arrangement of the reference inhibitor–donepezil [25]. Their basic moieties were close to the amino acid residues from the catalytic triad and anionic site. Alkyl linkers interacted with hydrophobic residues in the middle part of the active gorge, and the large, biphenyl moieties reached the peripheral anionic site (PAS) [26]. The most active inhibitors among the tested compounds, **5** (IC_50_ = 2.76 µM) and **21** (IC_50_ = 3.86 µM), were azepane derivatives. The top-ranked pose of **21** is shown in Figure 8. We noticed that the azepane ring mimicked both the benzyl substituent and the basic group of donepezil in the acetylcholinesterase active site. Heterocyclic rings of the tested compounds were close to His440, Trp84 and Phe330, where ionized amine groups participate in cation–π interactions. The azepane fragment formed optimal hydrophobic interactions with Trp84. Additionally, the protonated nitrogen atom of inhibitor **21** created a hydrogen bond with water 1159. This type of interaction was also observed in the case of the piperidine moiety of donepezil. Hydrophobic alkyl linkers of the active compounds interacted with aromatic rings of Phe330, Phe331 from the anionic site, and Tyr121, Tyr334 in the PAS. The hydroxyl moiety of Tyr121 took part in the formation of a hydrogen bond network, interacting with water molecule 1159, which had contact with the above-mentioned compound. Additionally, ligand **21** created a hydrogen bond between the oxygen atom from the ether linker and water 1254, similarly to the oxygen in the carbonyl group from the indanone ring of donepezil. This water molecule was further connected to the main chain of Phe288 from the acyl pocket, which could stabilize the inhibitor-protein complex. In the case of active ligands, biphenyl fragments were substituted with a linker in the *para* or *meta* position. Rigid, aromatic moieties were arranged between Phe290 from the acyl pocket and residues from the PAS: Tyr70, Tyr121 and Trp279, creating hydrophobic and aromatic interactions. We found that all of the potent compounds contained a six-carbon linker. Inactive derivatives with a five-carbon chain (**7** and **12**) showed a different binding mode in comparison to the active ligands. Additionally, non-significant inhibitory activity could be related to *ortho* substitution in the biphenyl fragment (**15**, **18**, **22**). These derivatives were also docked in a different manner compared to the donepezil pose. Their basic amine rings were close to the PAS amino acids, and biphenyl moieties were docked near the anionic site and catalytic triad residues (not shown).

#### 2.3.2. Molecular Docking to the Human Butyrylcholinesterase

**Figure 9:**
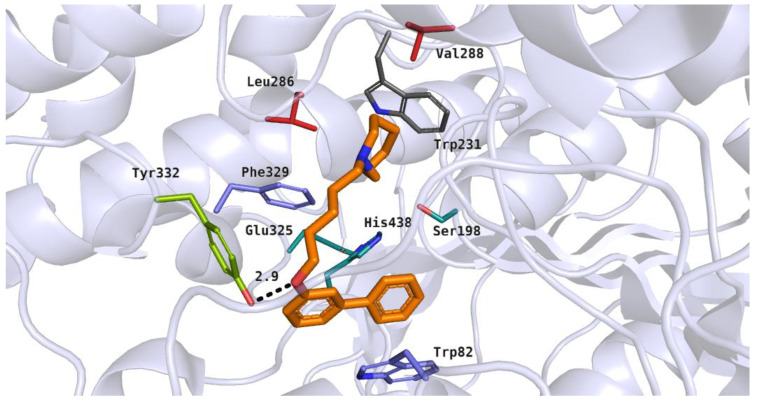
Binding mode of compound **20** within the butyrylcholinesterase active site. Residues colored in teal represent the catalytic triad, blue—anionic site, green—peripheral anionic site, red—acyl pocket.

The docking studies showed diverse binding modes for the tested compounds in the active site of human BuChE. For ligands with the *ortho* or *meta* substitution, the preferred binding mode was as follows: basic heterocyclic moieties were located between the catalytic triad and amino acid residues from the acyl pocket. Biphenyl fragments were close to the residues from the anionic site and alkyl linkers interacted with the acyl pocket and PAS residues. This binding mode was presented by the most active inhibitor, **20** (IC_50_ = 0.47 µM) (Figure 9). The heterocyclic moiety of compound **20** was docked near Ser198 from the catalytic triad and branched chain amino acids from the acyl pocket (Leu286 and Val288). The azepane fragment was able to participate in hydrophobic interactions with the above-mentioned residues and with the aromatic side chain of Trp231. The five-carbon alkyl linker was close to the aromatic residue Phe329 from the anionic site. The ether group formed a hydrogen bond with the hydroxyl moiety of Tyr332 from the peripheral anionic site. This type of interaction was noticed in our previous studies [17]. The flexible alkoxyl chain enabled the ligand to adopt a bent conformation. As a result of this, the large biphenyl fragment was close to the residues from the anionic site, participating in aromatic ring interactions with Trp82.

**Figure 10:**
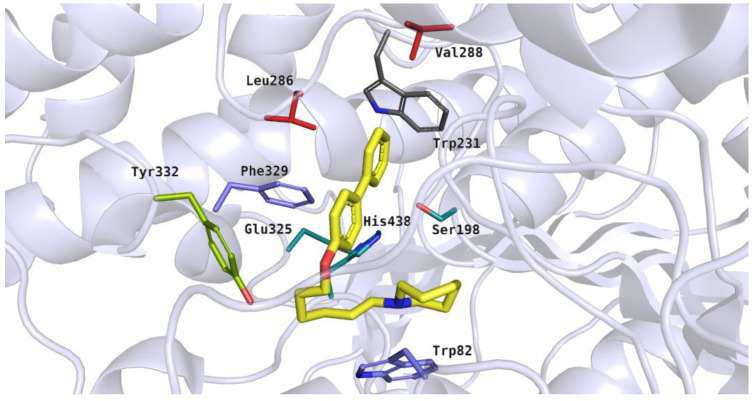
Binding mode of compound **5** within the butyrylcholinesterase active site. Residues colored in teal represent the catalytic triad, blue—anionic site, green—peripheral anionic site, red—acyl pocket.

Compounds with the *para*-substituted biphenyl moiety were bound within the BuChE active site in a different manner, compared to the *ortho-* or *meta*-substituted derivatives. This binding mode was presented by compound **5** (IC_50_ = 0.60 µM) (Figure 10). In this case, the heterocyclic moiety was observed close to the amino acid residues from the catalytic triad (His438) and anionic site (Trp82). The azepane ring underwent hydrophobic interactions and its protonated nitrogen atom was able to participated in a cation–π interaction with Trp82. A flexible tether allowed compound **5** to achieve bent conformation and fit to the enzyme active site. The rigid aromatic fragment interacted with hydrophobic amino acid residues from the acyl pocket (Leu286 and Val288) and anionic site (Phe329). Additionally, the biphenyl substituent participated in T-shaped aromatic interactions with the side chain of Trp231.

#### 2.3.3. Molecular Docking to the Human MAO B

**Figure 11:**
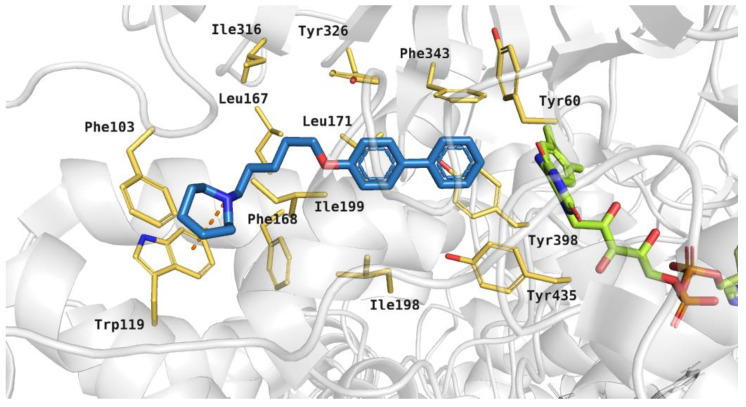
Binding mode of compound **19** within the *human* MAO B active site. Residues colored in yellow, green—cofactor FAD, orange dashes—cation-π interactions.

The tested compounds were docked to the *human* MAO B active site. The binding mode of the active compound **19,** which revealed significant *h*MAO B inhibitory activity with IC_50_ 243 nM, is shown in Figure 11. It was noticed that the azepane ring of the inhibitor participated in hydrophobic interactions with Phe103, Trp119, and Ile199, and a cation-π interaction between the protonated tertiary amine and aromatic ring of Trp119 was also observed. The five-carbon linker interacted with hydrophobic residues: Leu167 and Ile199. The large biphenyl fragment was located between the side chains of aromatic amino acids, such as: Tyr326, Phe343 and Tyr398. Additionally, this moiety of the ligand interacted with Leu171.

### 2.4. In Vivo Activity of Compound ***19***

Our previous in vitro studies [18] confirmed that **19** (**E153**) crosses the blood–brain barrier (BBB), showing typical effects for an H_3_R antagonist and low toxicity (hERG IC_50_ = 2.03 µM; hepatotoxicity IC_50_ = 12.8 µM; no mutagenic activity up to 10 µM). Moreover, **19** had selectivity towards other histamine receptors: H_1_ (*K_i_* = 1.3 µM) and H_4_ (*K_i_* = 21.8 µM) [18]. Additionaly, in silico ADMET properties calculated using server SwissADME [27] and ProTox-II [28], showed promising results, e.g., drug-likeness, BBB penetration or low toxicity.

**Figure 12:**
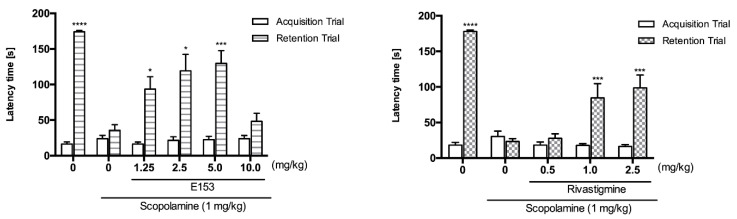
Activity of **19** (**E153**) and rivastigmine in the Passive Avoidance Task. **19** (**E153**) (1.25, 2.5, 5.0 or 10 mg/kg, i.p.) or rivastigmine (0.5, 1.0 or 2.5 mg/kg, i.p.) at the same volume of vehicle (1% Tween 80) was administered 30 min before the acquisition trial. Memory impairment was induced by scopolamine (1 mg/kg, i.p.) 20 min before the acquisition trial. 24 h after the acquisition trial, a retention trial was conducted for 180 s. Data represent means ± SEM (n = 7–9 per group); * *p*  <  0.05, *** *p*  <  0.001, **** *p*  <  0.0001 versus the scopolamine-treated group. Statistical analysis: two-way ANOVA followed by Tukey’s Multiple Comparison Test.

Taking into account previous and current in vitro results, we considered **19** as a promising subject for memory studies. As a comparator, we used rivastigmine, which is an acetylcholinesterase and butyrylcholinesterase inhibitor successfully used in the treatment of dementia. To evaluate the in vivo activity and confirm the potential of compound **19** to improve memory deficits, we tested it in the mouse model of scopolamine-induced memory impairments using a Passive Avoidance Task (PAT). This procedure was created for studying short-term memory as well as long-term memory. The paradigm requires the rodents to behave contrary to their innate preference for dark areas and for avoiding bright ones. This effect is obtained by applying non-harmful but aversive stimuli to mice, which induces signaling pathways for short- and long-term memory formation. We hypothesized that these mechanisms, impaired by scopolamine, would be (fully or partially) restored by the tested compound **19**. In the acquisition trial of the PAT, neither scopolamine, nor **19** nor rivastigmine significantly affected latency times. However, we observed significant differences in the retention trial (Figure 12). Scopolamine (1 mg/kg) compared with the control group treated with 1% tween and saline significantly reduced latency times (35.8 ± 7.8 vs. 174.00 ± 2.1; **** *p* < 0.0001). The memory impairment induced by scopolamine was significantly diminished by pretreatment with the tested compound **19**. Doses of 1.25 mg/kg, 2.5 mg/kg and 5.0 mg/kg increased the latency times to 93.5 ± 17.4, 119.1 ± 21.2, and 129.8 ± 17.8, respectively. The administration of a higher dose (10 mg/kg) resulted in lower activity of this compound. Rivastigmine was significantly active at doses of 1.0 mg/kg and 2.5 mg/kg, which increased the latency times to 84.6 ± 20.1 and 98.7 ± 17.9, respectively. There was no significant difference between these two doses, which shows that the activity of rivastigmine in the PAT is characterized by the “ceiling effect”. Moreover, we observed that the maximal value of latency time (129.8 s) in our experiments was reached after administration of **19** at the dose of 5.0 mg/kg. This result may suggest that the multidirectional mechanism of action of this compound (directly and indirectly affecting more neurotransmitters) may lead to more pronounced effects (better efficacy) in the reduction of memory impairment than a compound with a single mechanism. Thus, we can conclude that the pretreatment with **19** (before scopolamine on the first day of the experiment) affected the acquisition and at least the early consolidation phases, but not the retrieval phase of the memory formation.

## 3. Conclusions

To sum up, this report described promising biphenyl derivatives that are histamine H_3_R ligands with selective BuChE inhibition as potential compounds to efficiently treat AD. All compounds showed a preferential inhibition of BuChE over AChE, and most of them exhibited (sub)nanomolar inhibitory activity of BuChE. Pentyloxy derivatives selectively inhibited cholinesterases, whereas some hexyloxy derivatives also displayed weak AChE inhibition (IC_50_ ≥ 2.76 µM). Furthermore, the presence of an azepane moiety in the structure increased the inhibitory activity of compounds towards BuChE (IC_50_ ≤ 0.87 µM). Compound **19** (**E153**) proved to be the most promising, with a high affinity for *h*H_3_R (*K_i_* = 33.9 nM) and potent inhibition of BuChE (IC_50_ = 590 nM). Moreover, **19** (**E153**) showed inhibition of *h*MAO B with an IC_50_ of 243 nM. Furthermore, **19** (**E153**) reversed memory impairments induced by scopolamine in the Passive Avoidance Task. Thus, **19** (**E153**) can be a promising leading structure in the search for dual H_3_R-BuChE inhibitors as well as for multitarget inhibitors (H_3_R/BuChE/MAO B), as it shows a nanomolar concentration range affinity for all of the biological targets.

## 4. Materials and Methods

### 4.1. Chemistry

Reagents and solvents were purchased from commercial suppliers: Sigma Aldrich (Darmstadt, Germany) or Alfa Aesar (Karlsruhe, Germany) and were used without further purification. Melting points (m.p.) were determined in open capillaries on MEL-TEMP II apparatus (LD Inc. Long Beach, CA, USA) and are uncorrected. The NMR spectra (^1^H and ^13^C) were recorded in DMSO-d_6_ using Advance III HD 400 MHz spectrometer (Bruker, Billerica, MA, USA) or FT-NMR 500 MHz spectrometer (Joel Ltd., Akishima, Tokyo, Japan). The chemical shifts (*δ*) are reported in relation to tetramethylsilane (TMS) and the coupling constants (J) are expressed in Hz. The multiplicity of each peak is reported as: s, singlet; d, dublet; t, triplet; q, quartet; quin, quintet; m, multiplet; br, broad; def, deformed. NMR data of all new compounds are available in Supplementary Materials. Mass spectra (LC/MS) were performed on Waters TQ Detector Mass Spectrometer (Water Corporation, Milford, CT, USA). Retention times (t_R_) are given in minutes. UPLC/MS analysis confirmed purity of compounds (≥95%). The elemental analysis (C, H, N) was performed on Vario EL III Elemental Analyser (Hanau, Germany) and results agreed within 0.4% of the theoretical value. TLC data were obtained with Merck silica gel 60F_254_ aluminum sheets with the following detection with UV light and evaluation with Dragendorff’s reagent (solvent system: methylene chloride: methanol 9:1).

Synthesis of compounds **3–9**, **16**, **17**, **19–21** and starting materials-bromides: 3-((5-bromopentyl)oxy)-1,1′-biphenyl (CAS:1149748-49-1), 3-((6-bromohexyl)oxy)-1,1′-biphenyl, 4-((5-bromopentyl)oxy)-1,1′-biphenyl (CAS: 52273-19-5) and 4-((6-bromohexyl)oxy)-1,1′-biphenyl (CAS: 158136-46-0) was described previously [18]. 2-((6-Bromohexyl)oxy)-1,1′-biphenyl—a starting material for the synthesis of compounds **10**, **15**, **18** and **22** was prepared as described by Łażewska et al. [18].

#### 4.1.1. General Procedure of Synthesis of Compounds **10**–**15**, **18** and **22**

To the appropriate bromide (5 mmol) in the mixture of 105 mL ethanol, 20 mL water and 7.5 mmol K_2_CO_3_ (1.04 g) the appropriate amine (10 mmol) was added and the solution was refluxed for 15–20 h. After filtration of solid, the residue was evaporated and purified by extraction [18]. Oily product was crystallized with oxalic acid from diethyl ether/ethanol.

#### 4.1.2. 1-(6-[1,1′-Biphenyl]-2-yloxy)hexyl)piperidine Hydrogen Oxalate (**10**)

Yield: 39% (420 mg; MW = 427.52); m.p. 163–165 °C; ^1^H NMR (DMSO-d_6_, 400 MHz) δ: 7.46–7.54 (m, 2H), 7.41 (t, *J* = 7.43 Hz, 2H), 7.27–7.36 (m, 3H), 7.07–7.14 (m, 1H), 7.02 (t, *J* = 7.24 Hz, 1H), 3.94–4.05 (m, 2H), 2.76–3.19 (m, 4H), 1.17–1.82 (m, 16H); ^13^C NMR (101 MHz, DMSO-d_6_) δ: 165.0, 155.9, 138.7, 130.9, 130.4, 129.7, 129.3, 128.3, 127.2, 121.2, 113.2, 68.1, 56.3, 52.5, 28.8, 26.2, 25.5, 23.7, 23.1, 22.0; LC-MS: purity 96.18% t_R_ = 6.04, (ESI) *m*/*z* [M + H]^+^ 338.47; Anal. calcd. for C_25_H_33_NO_5_: C, 70.23; H, 7.78; N, 3.28%; Found: C, 70.41; H, 8.00; N, 3.18%.

#### 4.1.3. 1-(5-([1,1′-Biphenyl]-4-yloxy)pentyl)-3-methylpiperidine Hydrogen Oxalate (**11**)

Yield: 32% (690 mg; MW = 427.52); m.p. 143–146 °C; ^1^H NMR (500 MHz, DMSO-d_6_) δ: 7.51–7.60 (m, 4H), 7.39 (t, *J* = 7.59 Hz, 2H), 7.26 (def t, 1H), 6.97 (d, *J* = 8.59 Hz, 2H), 3.97 (t, *J* = 6.30 Hz, 2H), 3.21–3.39 (m, 2H), 2.94 (t, *J* = 7.88 Hz, 2H), 2.66 (t, *J* = 10.60 Hz, 1H), 2.41 (t, *J* = 11.60 Hz, 1H), 1.61–1.89 (m, 8H), 1.40 (t, *J* = 7.30 Hz, 2H), 0.95–1.08 (m, 1H), 0.84 (d, *J* = 6.59 Hz, 3H); ^13^C NMR (126 MHz, DMSO-d_6_) δ: 165.3, 158.8, 140.4, 133.0, 129.4, 128.3, 127.2, 126.7, 115.4, 67.7, 57.9, 56.4, 56.3, 56.3, 56.3, 52.0, 30.6, 29.0, 28.7, 23.5, 23.4, 22.7, 22.7, 19.1; LC-MS: purity 100% t_R_ = 6.13, (ESI) *m*/*z* [M + H]^+^ 338.36; Anal. calcd. for C_25_H_33_NO_5_: C, 70.23; H, 7.78; N, 3.28%; Found: C, 70.07; H, 8.01; N, 3.33%.

#### 4.1.4. 1-(5-([1,1′-Biphenyl]-3-yloxy)pentyl)-3-methylpiperidine Hydrogen Oxalate (**12**)

Yield: 36% (780 mg; MW = 427.52); m.p. 164–167 °C; ^1^H NMR (500 MHz, DMSO-d_6_) δ: 7.62 (d, *J* = 7.73 Hz, 2H), 7.38–7.47 (m, 2H), 7.32 (t, *J* = 7.73 Hz, 2H), 7.17 (d, *J* = 7.73 Hz, 1H), 7.13 (s, 1H), 6.89 (dd, *J* = 1.72, 8.02 Hz, 1H), 4.01 (t, *J* = 6.30 Hz, 2H), 3.19–3.37 (m, 2H), 2.94 (t, *J* = 7.88 Hz, 2H), 2.66 (t, *J* = 10.74 Hz, 1H), 2.40 (t, *J* = 11.74 Hz, 1H), 1.57–1.90 (m, 8H), 1.41 (quin, *J* = 7.45 Hz, 2H), 0.94–1.08 (m, 1H), 0.84 (d, *J* = 6.59 Hz, 3H); ^13^C NMR (126 MHz, DMSO-d_6_) δ: 165.3, 159.6, 142.2, 140.6, 130.5, 129.4, 128.1, 127.3, 119.5, 114.1, 113.2, 67.6, 57.9, 52.0, 30.6, 28.7, 23.5, 23.4, 19.1; LC-MS: purity 100% t_R_ = 6.00, (ESI) *m*/*z* [M + H]^+^ 338.47; Anal. calcd. for C_25_H_33_NO_5_: C, 70.23; H, 7.78; N, 3.28%; Found: C, 69.86; H, 7.41; N, 3.41%.

#### 4.1.5. 1-(6-([1,1′-Biphenyl]-4-yloxy)hexyl)-3-methylpiperidine Hydrogen Oxalate (**13**)

Yield 59% (1300 mg; MW = 441.55); m.p. 138–140 °C; ^1^H NMR (500 MHz, DMSO-d_6_) δ: 7.48–7.62 (m, 4H), 7.39 (t, *J* = 7.73 Hz, 2H), 7.21–7.30 (m, 1H), 6.97 (d, *J* = 8.59 Hz, 2H), 3.96 (t, *J* = 6.30 Hz, 2H), 3.18–3.37 (m, 2H), 2.91 (t, *J* = 8.02 Hz, 2H), 2.65 (t, *J* = 10.74 Hz, 1H), 2.40 (t, *J* = 11.74 Hz, 1H), 1.77–1.89 (m, 1H), 1.56–1.75 (m, 7H), 1.41 (quin, *J* = 7.45 Hz, 2H), 1.25–1.35 (m, 2H), 0.93–1.07 (m, 1H), 0.84 (d, *J* = 6.59 Hz, 3H); ^13^C NMR (126 MHz, DMSO-d_6_) δ: 165.3, 158.8, 140.4, 132.9, 129.4, 128.3, 127.2, 126.7, 115.4, 67.9, 57.9, 56.4, 56.4, 56.4, 51.9, 30.6, 29.0, 28.9, 26.4, 25.6, 23.7, 22.7, 22.7, 19.1; LC-MS: purity 98.24% t_R_ = 6.38, (ESI) *m*/*z* [M + H]^+^ 352.16; Anal. calcd. for C_26_H_35_NO_5_: C, 70.72; H, 7.99; N, 3.17%; Found: C, 70.77; H, 7.98; N, 3.15%.

#### 4.1.6. 1-(6-([1,1′-Biphenyl]-3-yloxy)hexyl)-3-methylpiperidine Hydrogen Oxalate (**14**)

Yield 40% (880 mg; MW = 441.55); m.p. 169–172 °C; ^1^H NMR (500 MHz, DMSO-d_6_) δ: 7.62 (d, *J* = 7.45 Hz, 2H), 7.38–7.46 (m, 2H), 7.29–7.35 (m, 2H), 7.17 (d, *J* = 7.73 Hz, 1H), 7.13 (d, *J* = 2.00 Hz, 1H), 6.89 (dd, *J* = 2.00, 8.02 Hz, 1H), 4.00 (t, *J* = 6.44 Hz, 2H), 3.18–3.37 (m, 2H), 2.91 (t, *J* = 7.73 Hz, 2H), 2.65 (t, *J* = 11.03 Hz, 1H), 2.40 (t, *J* = 11.60 Hz, 1H), 1.76–1.87 (m, 1H), 1.55–1.76 (m, 7H), 1.42 (quin, *J* = 7.52 Hz, 2H), 1.26–1.36 (m, 2H), 0.94–1.06 (m, 1H), 0.84 (d, *J* = 6.59 Hz, 3H); ^13^C NMR (126 MHz, DMSO-d_6_) δ: 165.1, 159.7, 142.2, 140.6, 140.4, 133.0, 130.5, 129.4, 128.3, 128.1, 127.4, 127.3, 126.7, 119.5, 115.4, 114.1, 113.2, 67.9, 67.8, 57.9, 57.9, 57.9, 57.9, 56.5, 56.4, 52.0, 30.6, 29.1, 29.1, 29.1, 29.1, 29.0, 28.9, 26.4, 25.6, 23.7, 22.8, 19.1; LC-MS: purity 96.16% t_R_ = 6.46, (ESI) *m*/*z* [M + H]^+^ 352.29; Anal. calcd. for C_26_H_35_NO_5_: C, 70.72; H, 7.99; N, 3.17%; Found: C, 70.48; H, 8.06; N, 3.15%.

#### 4.1.7. 1-(6-([1,1′-Biphenyl]-2-yloxy)hexyl)-3-methylpiperidine Hydrogen Oxalate (**15**)

Yield 36% (400 mg; MW = 441.55); m.p. 135–137 °C (EtOH); ^1^H NMR (400 MHz, DMSO-d_6_) δ: 7.46–7.53 (m, 2H), 7.41 (t, *J* = 7.63 Hz, 2H), 7.25–7.36 (m, 3H), 7.10 (d, *J* = 8.22 Hz, 1H), 7.02 (t, *J* = 7.43 Hz, 1H), 3.97 (t, *J* = 6.26 Hz, 2H), 3.21–3.43 (m, 2H), 2.81–3.00 (m, 2H), 2.62–2.75 (m, 1H), 2.43 (t, *J* = 11.74 Hz, 1H), 1.53–1.97 (m, 8H), 1.18–1.44 (m, 4H), 1.00–1.15 (m, 1H), 0.89 (d, *J* = 6.65 Hz, 3H); ^13^C NMR (101 MHz, DMSO-d_6_) δ: 165.1, 155.9, 138.7, 130.9, 130.4, 129.7, 129.3, 128.3, 127.2, 121.2, 113.2, 68.1, 57.9, 51.9, 30.6, 28.8, 26.2, 25.5, 23.6, 19.1; LC-MS: purity 100% t_R_ = 6.27, (ESI) *m*/*z* [M + H]^+^ 352.49; Anal. calcd. for C_26_H_35_NO_5_: C, 70.72; H, 7.99; N, 3.17%; Found: C, 70.81; H, 8.11; N, 3.17%.

#### 4.1.8. 1-(6-([1,1′-Biphenyl]-2-yloxy)hexyl)-4-methylpiperidine Hydrogen Oxalate (**18**)

Yield 8% (85 mg; MW = 441.55); m.p. 184–186 °C (EtOH); ^1^H NMR (400 MHz, DMSO-d_6_) δ: 7.46–7.55 (m, 2H), 7.41 (t, *J* = 7.43 Hz, 2H), 7.26–7.36 (m, 3H), 7.10 (d, *J* = 8.22 Hz, 1H), 7.02 (t, *J* = 7.43 Hz, 1H), 3.97 (t, *J* = 6.26 Hz, 2H), 3.24–3.40 (m, 2H), 2.73–2.98 (m, 4H), 1.76 (d, *J* = 13.30 Hz, 2H), 1.54–1.69 (m, 5H), 1.20–1.45 (m, 6H), 0.92 (d, *J* = 6.26 Hz, 3H); ^13^C NMR (101 MHz, DMSO-d_6_) δ: 165.1, 155.9, 138.7, 130.9, 130.4, 129.7, 129.3, 128.3, 127.2, 121.2, 113.3, 68.1, 56.0, 51.8, 31.0, 28.8, 28.5, 26.2, 25.5, 23.7, 21.3, 19.0; LC-MS: purity 95.78% t_R_ = 6.37, (ESI) *m*/*z* [M + H]^+^ 352.49; Anal. calcd. for C_26_H_35_NO_5_: C, 70.72; H, 7.99; N, 3.17%; Found: C, 70.34; H, 8.18; N, 3.15%.

#### 4.1.9. 1-(6-([1,1′-Biphenyl]-2-yloxy)hexyl)azepane Hydrogen Oxalate (**22**)

Yield 35% (390 mg; MW = 441.55); m.p. 146–148 °C; ^1^H NMR (400 MHz, DMSO-d_6_) δ: 7.47–7.55 (m, 2H), 7.41 (t, *J* = 7.43 Hz, 2H), 7.26–7.36 (m, 3H), 7.10 (d, *J* = 7.82 Hz, 1H), 7.02 (t, *J* = 7.43 Hz, 1H), 3.86–4.06 (m, 2H), 3.18 (br. s., 4H), 2.89–3.04 (m, 2H), 1.78 (br. s., 4H), 1.51–1.67 (m, 8H), 1.19–1.43 (m, 4H); ^13^C NMR (101 MHz, DMSO-d_6_) δ: 165.1, 155.9, 138.7, 130.9, 129.7, 129.3, 128.3, 127.2, 121.2, 113.2, 68.1, 56.7, 53.9, 28.8, 26.5, 26.2, 25.5, 24.0, 23.5; LC-MS: purity 95.21% t_R_ = 6.27, (ESI) *m*/*z* [M + H]^+^ 352.49; Anal. calcd. for C_26_H_35_NO_5_: C, 70.72; H, 7.99; N, 3.17%; Found: C, 70.76; H, 8.20; N, 3.10%.

### 4.2. In Vitro Biological Studies

#### 4.2.1. Evaluation of Histamine H_3_ Receptor Affinity

Affinity to human histamine H_3_ receptor stably expressed in HEK293 cells was evaluated in a radioligand binding assay with *N^α^*-methylhistamine according to the previous procedure [18,21]. Non-specific binding was defined with presence of 10 mM pitolisant. The bound radioligand was separated from the free radioligand by filtration through GF/B filters (pretreated with 0.3% (*m*/*v*) polyethylenimine) using Inotech cell harvester (Dottikin, Switzerland). Unbound radioligand was removed by three washing steps with ice-cold water. The radioactivity was determined in a liquid scintillation counter (Perkin Elmer Trilux Betacounter, Germany). Scintillation data corrected for non-specific binding were analyzed using GraphPad Prism (V6.01, San Diego, CA, USA) software. K_i_ values were calculated from IC_50_ values (from at least three experiments, performed in duplicates) according to the Cheng–Prusoff equation [29].

#### 4.2.2. Evaluation of Cholinesterase Inhibitory Activity

Inhibitory activities of the tested compounds against cholinesterases were measured using a spectrophotometric method modified for 96-well microplates as described recently [17]. Enzymes (AChE from *electrophorus electricus*, BuChE from equine serum) and substrates (5,5′-dithiobis(2-nitrobenzoic acid, acetylthiocholine iodide and butyrylthiocholine iodide) were purchased from Sigma Aldrich. Donepezil and rivastigmine were used as the reference compounds

Briefly, the enzymes (*ee*AChE, *eq*BuChE) were diluted with demineralized water to obtain final concentration of 0.384 U/mL. Tested compounds were prepared in DMSO and diluted with water to get desired concentration. First, all compounds were screened at the concentration of 10 μM. The changes in absorbance were measured at 412 nm, using a microplate reader (EnSpire Multimode; PerkinElmer, Waltham, MA, USA). Then, the percent of inhibition of tested enzymes for each compound was calculated. For compounds with higher than 50% of inhibitory activity IC_50_ values were determined using seven different concentrations of tested compound. Each concentration was measured in triplicate. All calculations were made using nonlinear regression GraphPad Prism 5 (GraphPad Software, San Diego, CA, USA).

#### 4.2.3. Kinetic Studies of eqBuChE Inhibition

The kinetic analysis was performed for compounds **5** and **19**, following the Ellman’s procedure [22], modified for 96-well microplates. *Eq*BuChE stock solution was diluted with demineralized water prior to use, giving the final concentration of 0.027 U/mL in the wells. Aqueous substrate BTC stock solution was also diluted before use to give six final concentrations in the wells: 0.3, 0.24, 0.18, 0.12, 0.06, and 0.04 mM. Six different concentrations of inhibitor were used to obtain enzyme activities between 30% and 80%. For each concentration of the target compound, all six BTC solutions were utilized to perform the assays. V_max_ and K_m_ values of the Michaelis−Menten kinetics were calculated by nonlinear regression from substrate−velocity curves. Lineweaver-Burk and Cornish-Bowden plots were calculated using linear regression in GraphPad Prism 5 (GraphPad Software, San Diego, CA, USA). Each experiment was performed in triplicate.

#### 4.2.4. Evaluation of Monoamine Oxidase Inhibitory Activity

Inhibition of *h*MAO B was evaluated using fluorometric Monoamine Oxidase assay as described by Łażewska et al. [24]. Recombinant MAO B was purchased from Sigma Aldrich (M7441). As the references were used safinamide (1 μM; Sigma Aldrich) and pargyline (10 µM; Sigma Aldrich). The assay was carried out in 96-well plate. Tested compounds dissolved in DMSO (1 μM) were added to wells that contained 98 µL of enzyme dilution (1 µg/well) in phosphate buffer (50 mM, pH 7.4). After the 30 min of preincubation at room temperature Amplex^TM^ Red (50 µL) and horse radish peroxidase (4 U/mL) were added. An enzymatic reaction started following addition of *para*-tyramine solution (200 μM). The signal was measured after 1h (excitation at 570 nm and emission at 585 nm) using EnSpire^®^ multimode plate reader (Perkin Elmer, Inc.). Compounds that inhibited the enzyme by more than 50% of pargyline were chosen for IC_50_ evaluation. Each experiment was performed in duplicate.

### 4.3. Molecular Docking to the Electric Ray Acetylcholinesterase, the Human Butyrylcholinesterase and the Human Monoamine Oxidase B

Docking studies were performed according to the previously validated procedures [24,26]. All of the tested compounds were prepared in Corina Classic software. Hydrogen atoms were added and all possible stereoisomers were included. Their ionization states were assigned at physiological pH with the Open Babel suite. Ligands were saved in mol2 format. Protein crystal structures were downloaded from Protein Data Bank. Enzyme structures (AChE, PDB code: 1EVE, BuChE, PDB code: 1P0I, and MAO B, PDB code: 4A79) were prepared as follow: the hydrogen atoms were added, all histidine residues were protonated at Nε atoms. Ligand and water molecules were removed, except water molecules 1159, 1249 and 1254 in AChE. As the crystal structure of horse BuChE is not available, we have chosen the human one because of high degree of sequence similarity and conservation of all crucial amino acid residues in the active site. Binding sites were defined as amino acid residues within the radius of 10 Å, 20 Å, and 12 Å from ligands: donepezil for AChE, glycerol for BuChE, and pioglitazone in case of MAO B. Docking studies were performed for each enzyme with GOLD 5.1 software. Standard parameters of genetic algorithm with population size 100 and operation number 100,000 were applied. For each ligand 10 poses were generated and sorted according to the ChemScore value. The results were analyzed in PyMOL software.

### 4.4. In Vivo Studies of Compound ***19***

#### 4.4.1. Animals

The experiments were carried out on adult male Albino Swiss mice (CD-1, 18–25 g). Animals were housed in plastic cages in room at a constant temperature of 20 ± 2°C, under light/dark (12:12) cycle and had free access to standard pellet diet and water. Experimental groups consisted of 6–12 animals, all the animals were used only once and they were killed by cervical dislocation immediately after the assay. Behavioral measures were scored by trained observers, which were blind to experimental conditions. Treatment of laboratory animals in the present study was in full accordance with the respective Polish regulations. 

#### 4.4.2. Compound **19** and Reference Compounds

For behavioral experiment, compound **19**, rivastigmine and scopolamine were suspended in 1% aqueous solution of Tween 80 and administered by the intraperitoneal (*i.p.*) route 30 min before the test. Control animals (negative control) were given an appropriate amount of vehicle (1% aqueous solution of Tween 80; i.p.) 30 min before the test.

#### 4.4.3. Step-Through Passive Avoidance Task

The task was performed according to the method described previously [30]. Briefly, the test was conducted in the apparatus with two compartments (LE872, Bioseb, France). The illuminated white compartment (20 cm × 21 cm × 20 cm, 1000 lx) was separated by an automated sliding gate with the smaller dark compartment (7.3 cm × 7.5 cm × 14 cm, 10 lx) equipped with an electric grid floor. For acquisition session, mice were first placed individually in the white compartment and after 30 s the gate to the smaller compartment was opened. Immediately after entering into the dark compartment, the gate was closed and the mouse was punished by an unavoidable electric foot shock (0.8 mA for 2 s). The mice which did not enter the dark compartment within 60 s were excluded from the study. Next day (24 h later), the pretested animals were placed again into the bright compartment. The experimental procedure was similar to acquisition session, but this time mice did not receive the electric shock after the entrance to the dark compartment. The latency time to cross through the gate between the compartments was measured. Mice, which avoided the dark compartment for 180 s were considered to remember the task.

## Figures and Tables

**Table 1:**
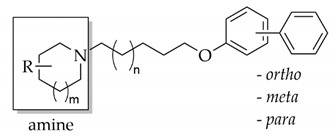
Structures and biological activities of tested compounds.

Comp.	Cyclic Amine	n	Substitution	*h*H_3_R ^a^ *K*_i_ [nM] Mean ± SD [CI 95%]	*ee*AChE ^b^ IC_50_ [μM] Mean ± SEM or (% Inh.) ^e^	*eq*BuChE ^c^ IC_50_ [μM] Mean ± SEM	*h*MAO B ^d^ IC_50_ [nM] Mean ± SEM or (%Inh.) ^f^
**6**	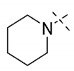	1	*para*	25.2 ± 9.3 ^g^	(24% ± 1)	2.16 ± 0.03	(50% ± 2)
**7**		1	*meta*	76.0 ± 9.2 ^g^	(24% ± 1)	1.29 ± 0.02	(11% ± 1)
**8**		2	*para*	91.5 ± 14.4 ^g^	(48% ± 1)	1.63 ± 0.05	(36% ± 13)
**9**		2	*meta*	149 ± 108 ^g^	(35% ± 2)	1.78 ± 0.08	(16% ± 0)
**10**		2	*ortho*	221 [23;2171]	(8% ± 2)	1.28 ± 0.02	(40% ± 3)
**11**	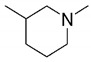	1	*para*	27.3 ± 15.1	(45% ± 3)	0.91 ± 0.01	(28% ± 3)
**12**		1	*meta*	45.0 ± 13.4	(27% ± 1)	0.68 ± 0.01	(5% ± 1)
**13**		2	*para*	146.5 ± 65.3	5.02 ± 0.09	0.95 ± 0.00	(20% ± 1)
**14**		2	*meta*	27 [17;43]	4.71 ± 0.13	1.52 ± 0.04	(35% ± 6)
**15**		2	*ortho*	23 [2;223]	(24% ± 1)	2.10 ± 0.06	(16% ± 1)
**3**	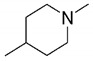	1	*para*	88.6 ± 31.1 ^g^	(42% ± 1) ^i^	0.85 ± 0.02 ^i^	(36% ± 2)
**16**		1	*meta*	157.0 ± 19.2 ^g^	(41% ± 1)	1.46 ± 0.03	(7% ± 1)
**4**		2	*para*	265.9 ± 83.7 ^g^	4.46 ± 0.10 ^i^	0.74 ± 0.02 ^i^	(27% ± 3)
**17**		2	*meta*	172 ± 73 ^g^	(48% ± 1)	1.49 ± 0.04	(5% ± 1)
**18**		2	*ortho*	561 [192;1638]	(20% ± 3)	1.48 ± 0.04	(24% ± 1)
**19 (E153)**	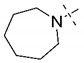	1	*para*	33.9 ± 8.1 ^g^	(44% ± 1)	0.59 ± 0.01	243 ± 73 (54% ± 1)
**20**		1	*meta*	114.7 ± 22.0 ^g^	(26% ± 3)	0.47 ± 0.01	(14% ± 0)
**5**		2	*para*	150.9 ± 72.8 ^g^	2.76 ± 0.04 ^i^	0.60 ± 0.01 ^i^	(45% ± 1)
**21**		2	*meta*	19 ± 5.5 ^g^	3.86 ± 0.08	0.87 ± 0.00	(43% ± 5)
**22**		2	*ortho*	528 [328;849]	(30% ± 5)	0.69 ± 0.05	(17% ± 0)
Pitolisant				12 ± 3 ^i^	(3% ± 1) ^i^	8.42 ± 0.18 ^i^	(2% ± 3)
Donepezil				Nt ^h^	0.041 ± 0.006 ^i^ 0.027 ± 0.002 ^j^	1.83 ± 0.017 ^i^	Nt ^h^
Rivastigmine				Nt ^h^	71 ± 3 ^j^	2.20 ± 0.05	Nt ^h^
Safinamide				Nt ^h^	Nt ^h^	Nt ^h^	7.7 ± 1.2

^a^ [^3^H]*N ^α^-*Methylhistamine binding assay in HEK293 cells stably expressing the human H_3_R; mean value ± standard deviation of triplicate independent experiments or within the 95% confidence interval (CI); ^b^ AChE from *electric eel*; IC_50_, mean value for three experiments; ^c^ BuChE from *equine serum*; IC_50_, mean value for three experiments; ^d^ Fluorometric MAO assay; mean values of two independent experiments. ^e^ % of inhibition of *ee*AChE at 10 μM; ^f^ % of inhibition of *h*MAO B at 1 μM. ^g^ data from [18]; ^h^ Nt, not tested; ^i^ data from [17]; ^j^ data from [19].

## Data Availability

Not applicable.

## Supplementary Materials

The following are available online. Spectral information (^1^H-NMR, ^13^C-NMR and LC-MS) of synthesized compounds.

## Sample Availability

Samples of the tested compounds are available from the authors.
