# Supplementary material for: Biphenylalkoxyamine Derivatives–Histamine H3 Receptor Ligands with Butyrylcholinesterase Inhibitory Activity
Source: Molecules. 2021 Jun 11;26(12):3580. doi: 10.3390/molecules26123580 (PMC8231170; doi:10.3390/molecules26123580)
Supplement: Supplementary file 1 [file molecules-26-03580-s001.zip › molecules-1247533-supplementary.pdf]

Biphenylalkoxyamine derivatives – histamine H_3 receptor ligands with butyrylcholinesterase inhibitory activity

Dorota Łażewska[1*], Paula Zaręba[2], Justyna Godyń[2], Agata Doroz-Płonka[1], Annika Frank[3], David Reiner-Link[3], Marek Bajda[2], Dorota Stary[2], Szczepan Mogilski[4], Agnieszka Olejarz-Maciej[1], Maria Kaleta[1], Holger Stark[3], Barbara Malawska[2], Katarzyna Kieć-Kononowicz[1]

[1] Department of Technology and Biotechnology of Drugs, Jagiellonian University Medical College, Medyczna Str. 9, 30-688 Kraków, Poland; a.doroz-plonka@uj.edu.pl (A.D.-P.); agnieszka.olejarz@uj.edu.pl (A.O.-M.); maria.kaleta@uj.edu.pl (M.K.); mfkonono@cyf-kr.edu.pl K.K.-K.)

[2] Department of Physicochemical Drug Analysis, Jagiellonian University Medical College, Medyczna Str. 9, 30-688 Kraków, Poland; paula.zareba@uj.edu.pl (P.Z.); justyna.godyn@uj.edu.pl (J.G.); marek.bajda@uj.edu.pl (M.B.); dorota.stary@gmail.com (D.S.); mfmalaws@cyf-kr.edu.pl (B.M.)

[3] Institute of Pharmaceutical and Medicinal Chemistry, Heinrich Heine University Düsseldorf, Universitaetsstr. 1, 40225 Duesseldorf, Germany; annikafrank@gmx.de (A.F.); david.reiner@sund.ku.dk (D.R.-L.); stark@hhu.de (H.S.)

[4] Department of Pharmacodynamic, Faculty of Pharmacy, Jagiellonian University Medical College, Medyczna Str. 9, 30-688 Kraków, Poland; szczepan.mogilski@uj.edu.pl (S.M.)

*Corresponding author: dlazewska@cm-uj.krakow.pl (D.Ł.)

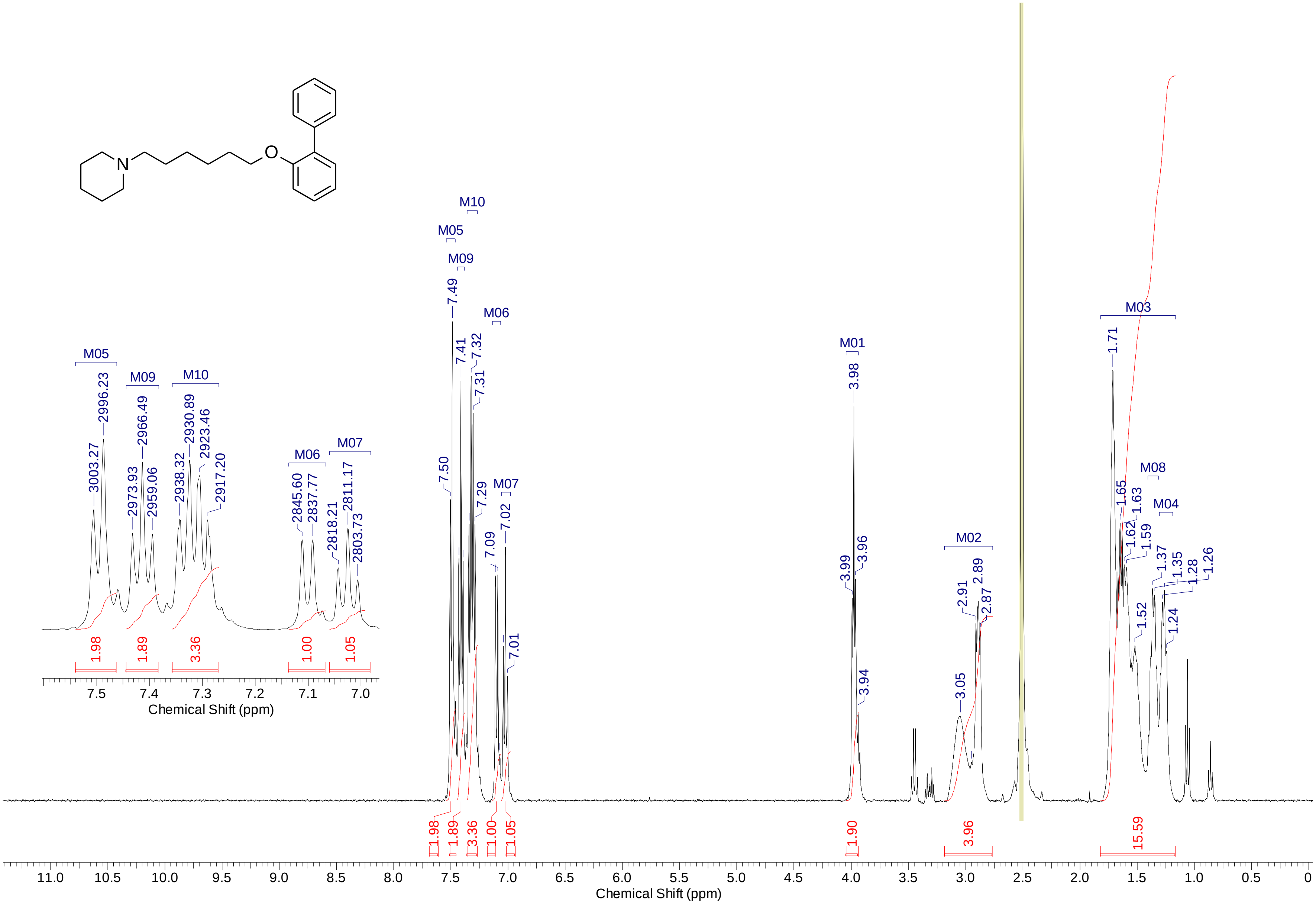

Figure S1. ^{1}H NMR spectrum of 1-(6-[1,1′-biphenyl]-2-yloxy)hexyl)piperidine hydrogen oxalate (**10**).

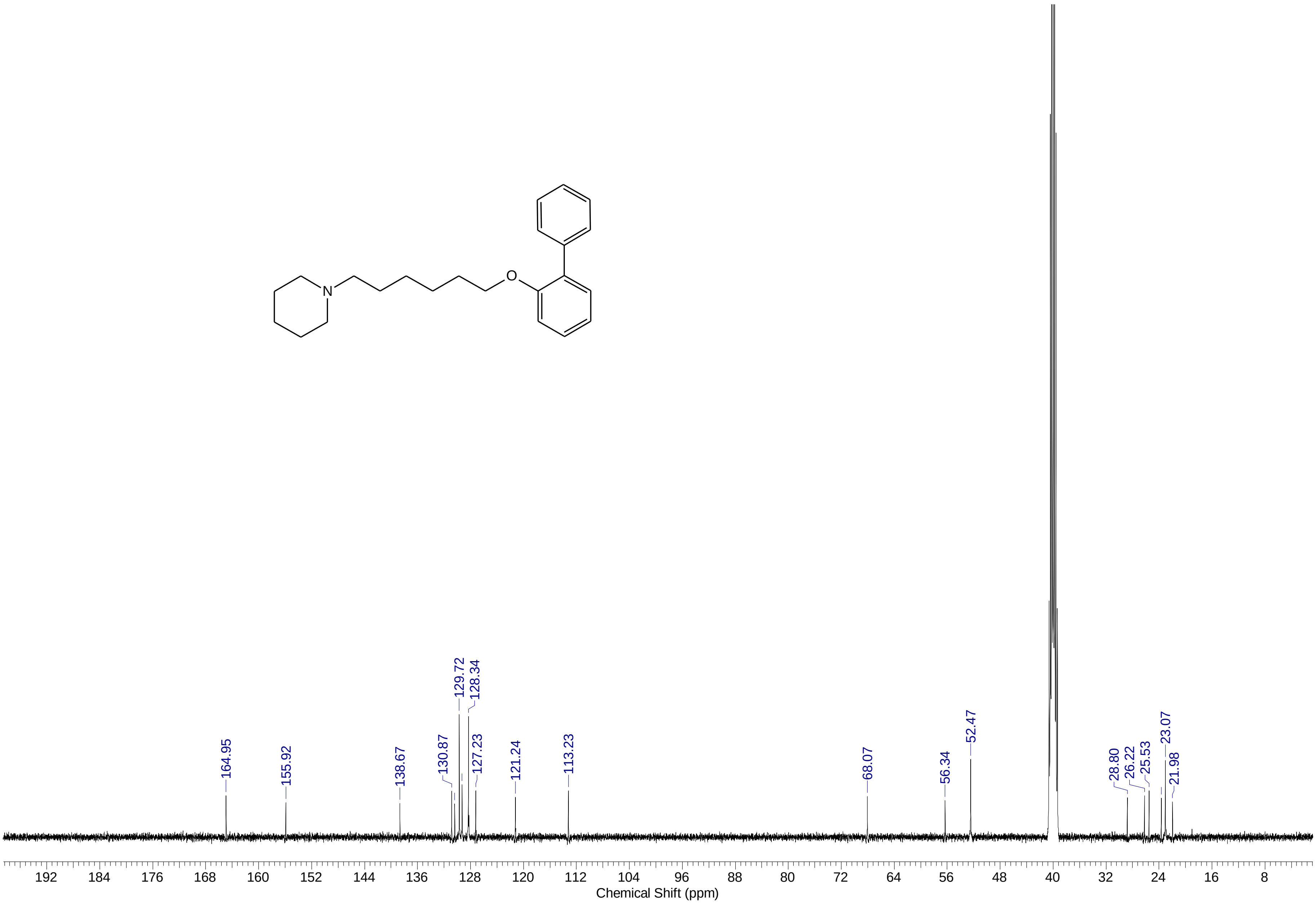

Figure S2. ^{13}C NMR spectrum of 1-(6-[1,1′-biphenyl]-2-yloxy)hexyl)piperidine hydrogen oxalate (**10**).

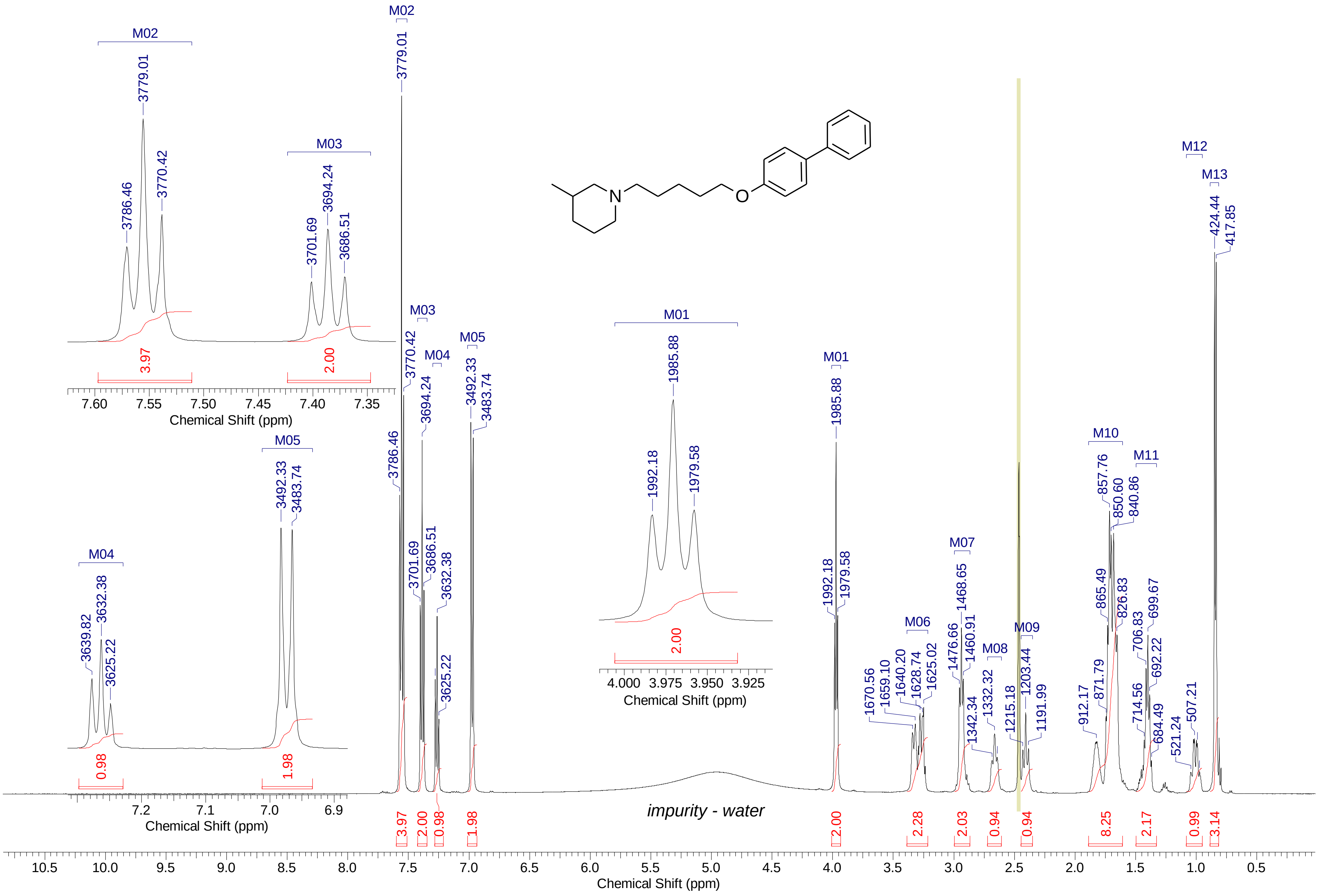

Figure S3. ^{1}H NMR spectrum of 1-(5-([1,1′-biphenyl]-4-yloxy)pentyl)-3-methylpiperidine hydrogen oxalate (**11**).

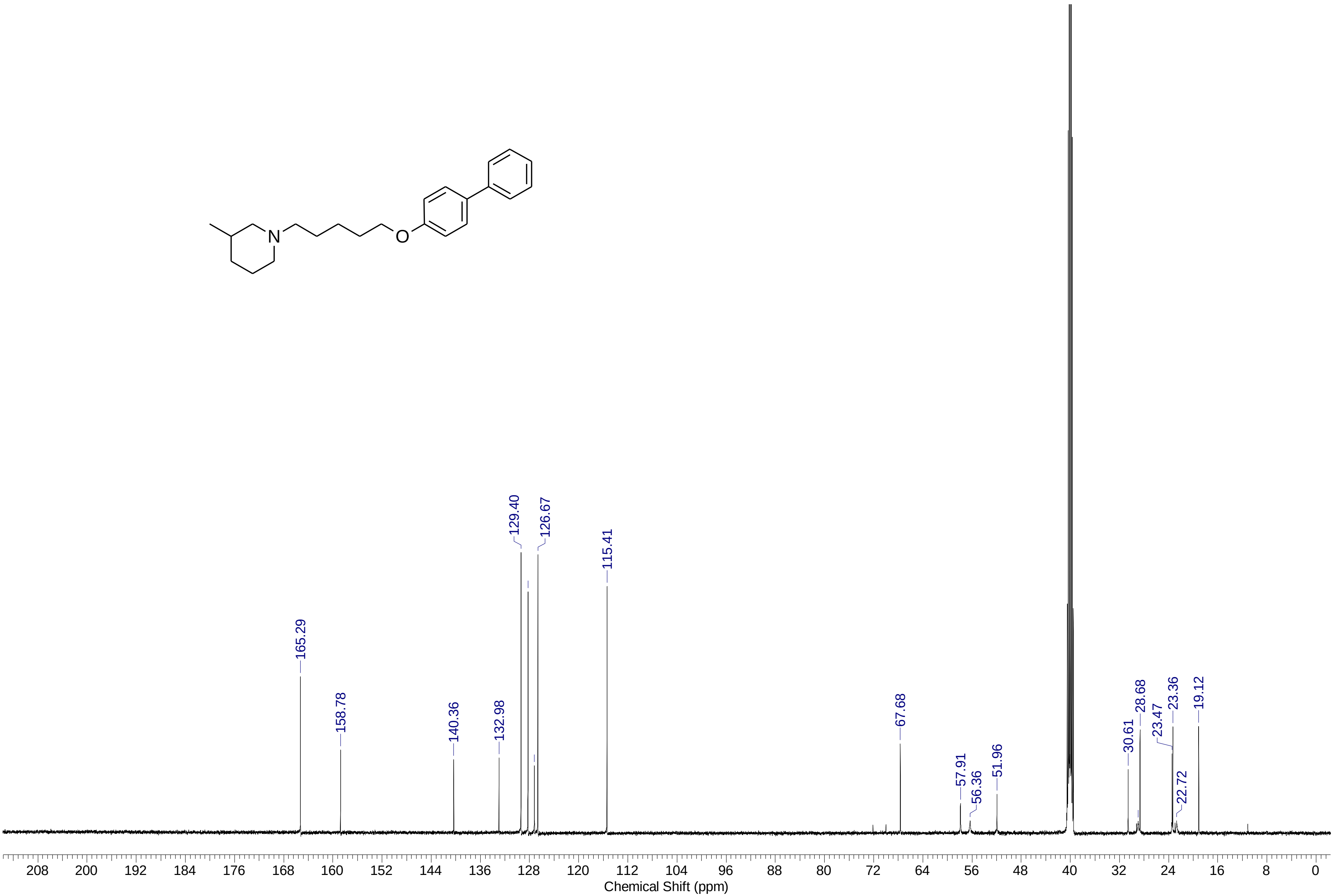

Figure S4. ^{13}C NMR spectrum of 1-(5-([1,1′-biphenyl]-4-yloxy)pentyl)-3-methylpiperidine hydrogen oxalate (**11**).

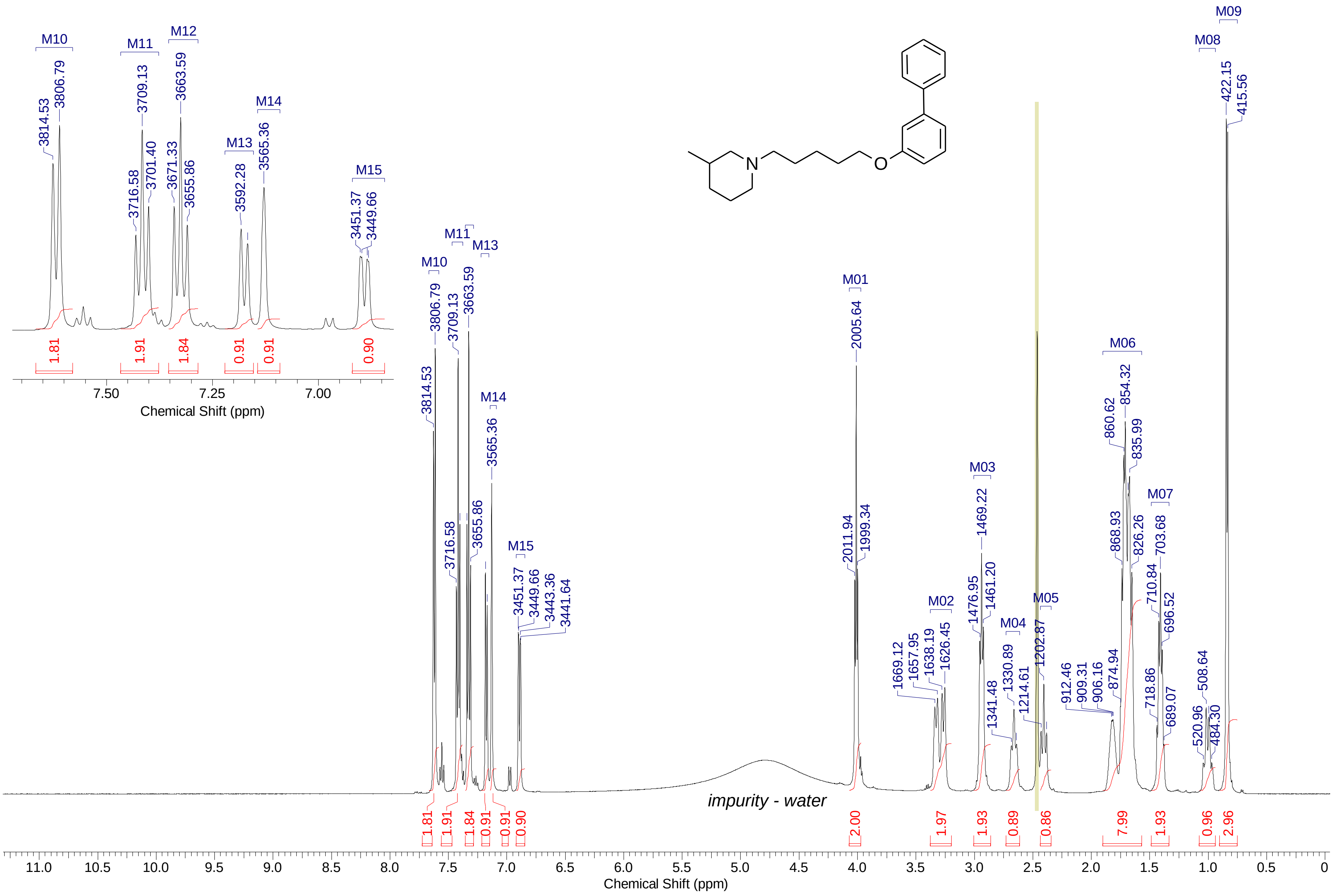

Figure S5. ^{1}H NMR spectrum of 1-(5-([1,1′-biphenyl]-3-yloxy)pentyl)-3-methylpiperidine hydrogen oxalate (**12**).

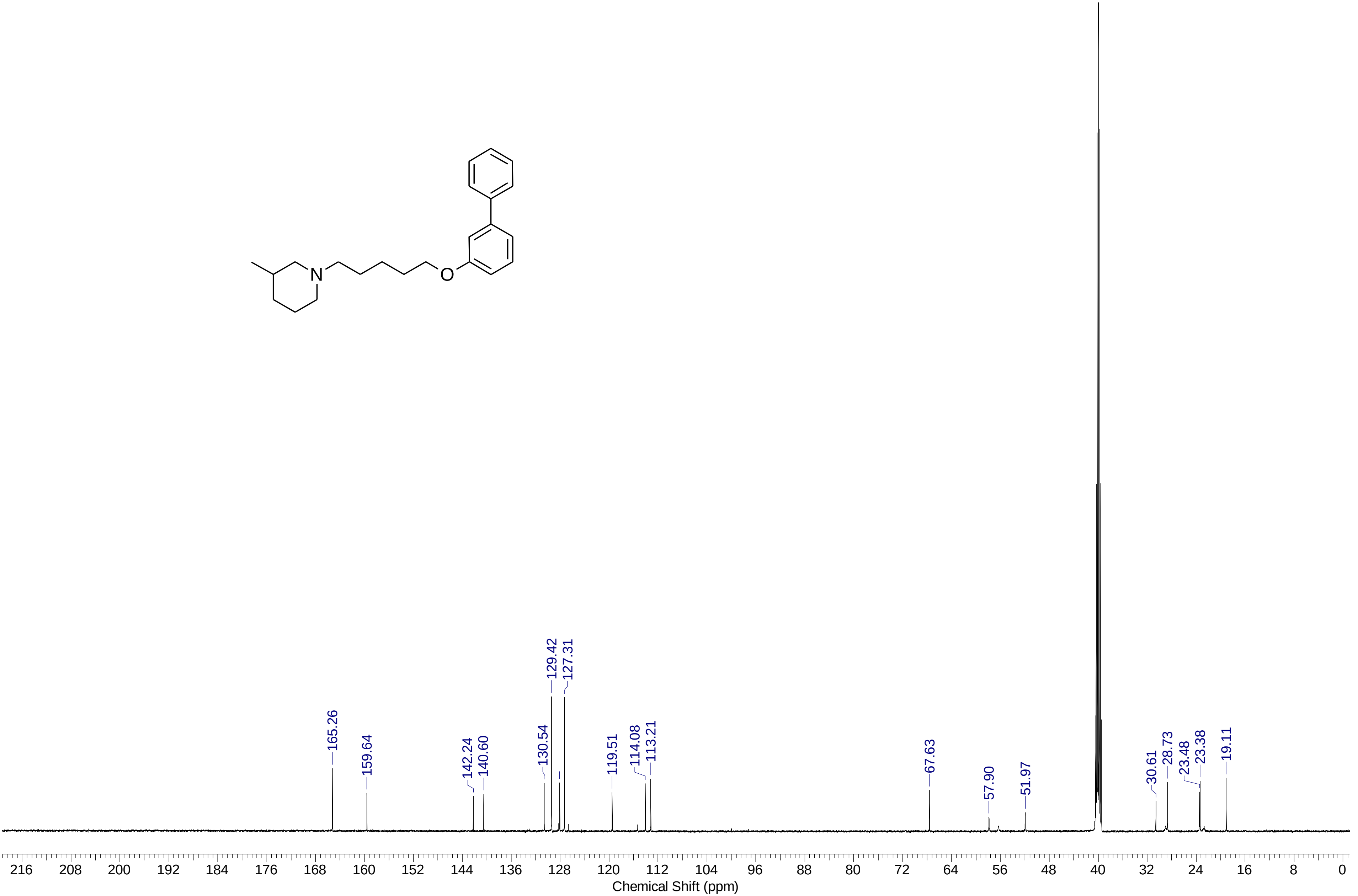

Figure S6. ^{13}C NMR spectrum of 1-(5-([1,1′-biphenyl]-3-yloxy)pentyl)-3-methylpiperidine hydrogen oxalate (**12**).

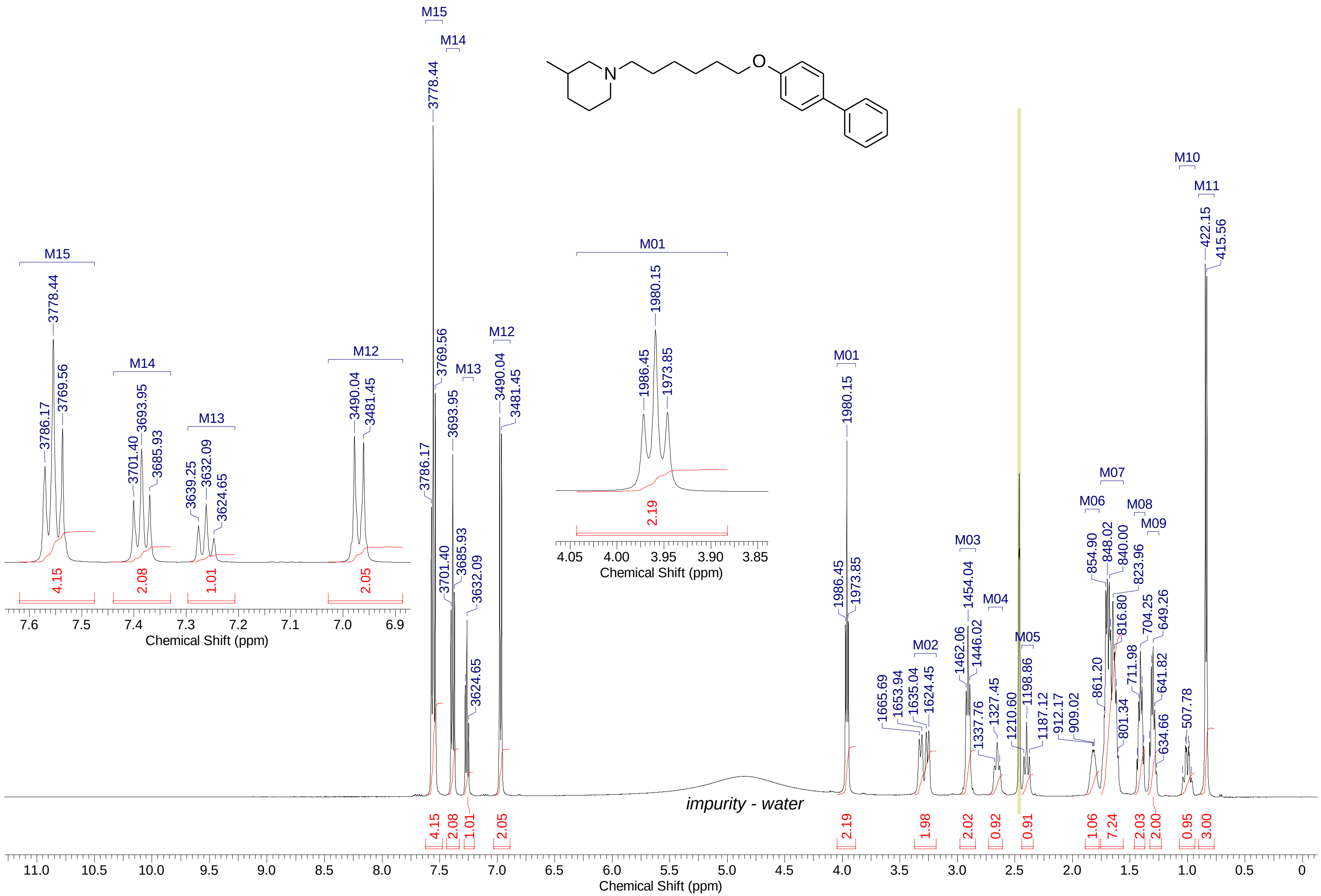

Figure S7. ^{1}H NMR spectrum of 1-(6-([1,1′-biphenyl]-4-yloxy)hexyl)-3-methylpiperidine hydrogen oxalate (**13**).

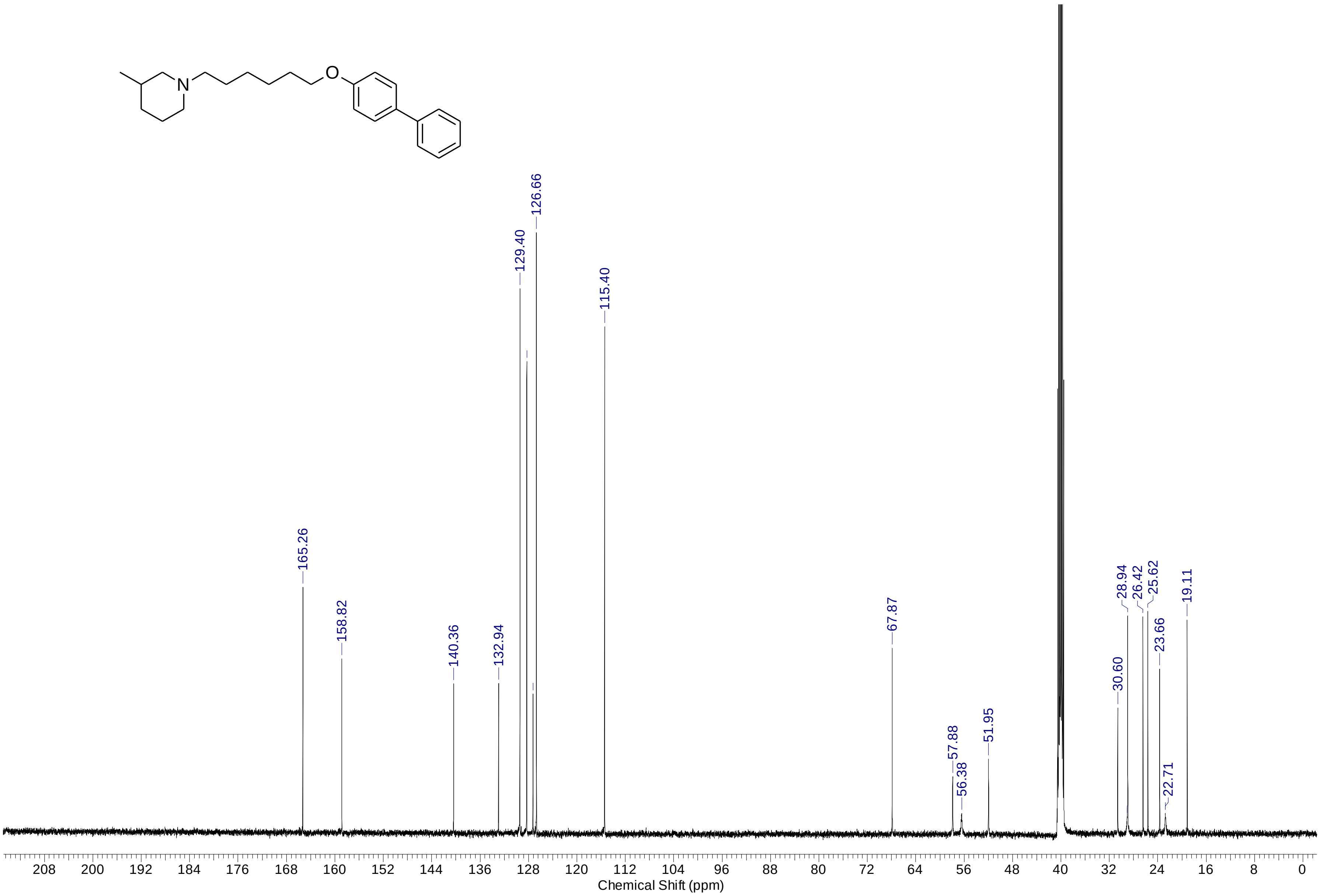

Figure S8. ^{13}C NMR spectrum of 1-(6-([1,1′-biphenyl]-4-yloxy)hexyl)-3-methylpiperidine hydrogen oxalate (**13**).

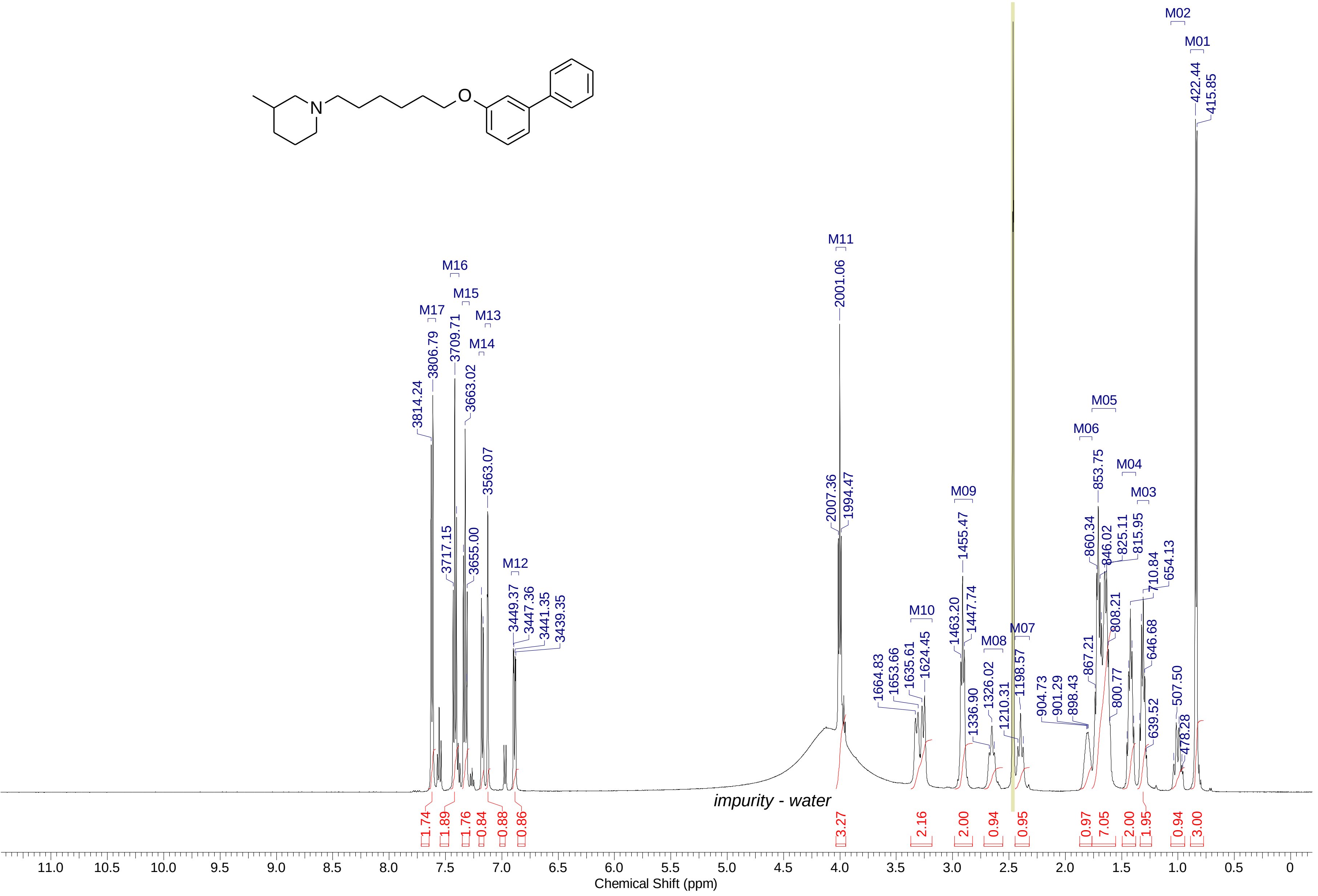

Figure S9. ^{1}H NMR spectrum of 1-(6-([1,1′-biphenyl]-3-yloxy)hexyl)-3-methylpiperidine hydrogen oxalate (**14**).

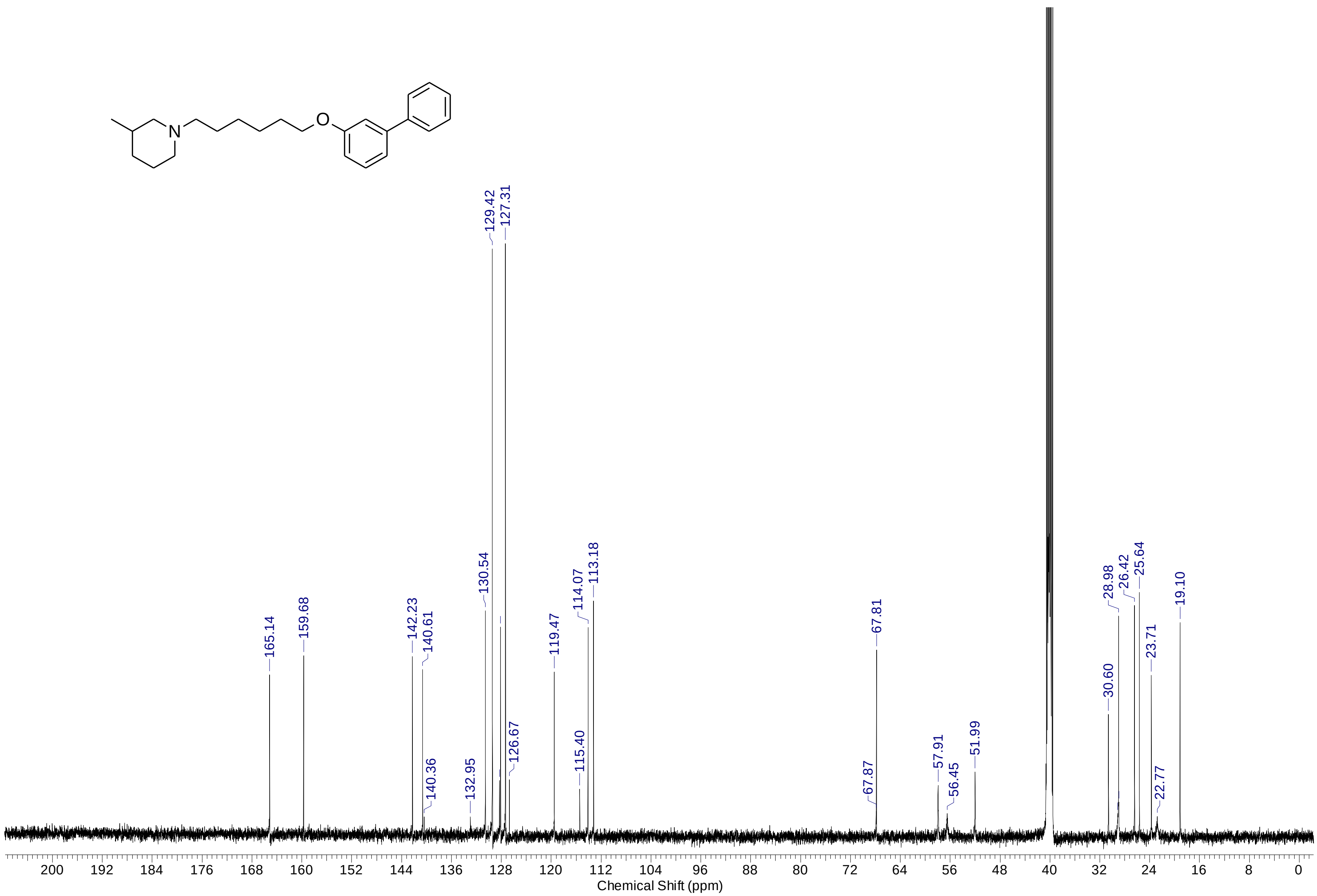

Figure S10. ^{13}C NMR spectrum of 1-(6-([1,1′-biphenyl]-3-yloxy)hexyl)-3-methylpiperidine hydrogen oxalate (**14**).

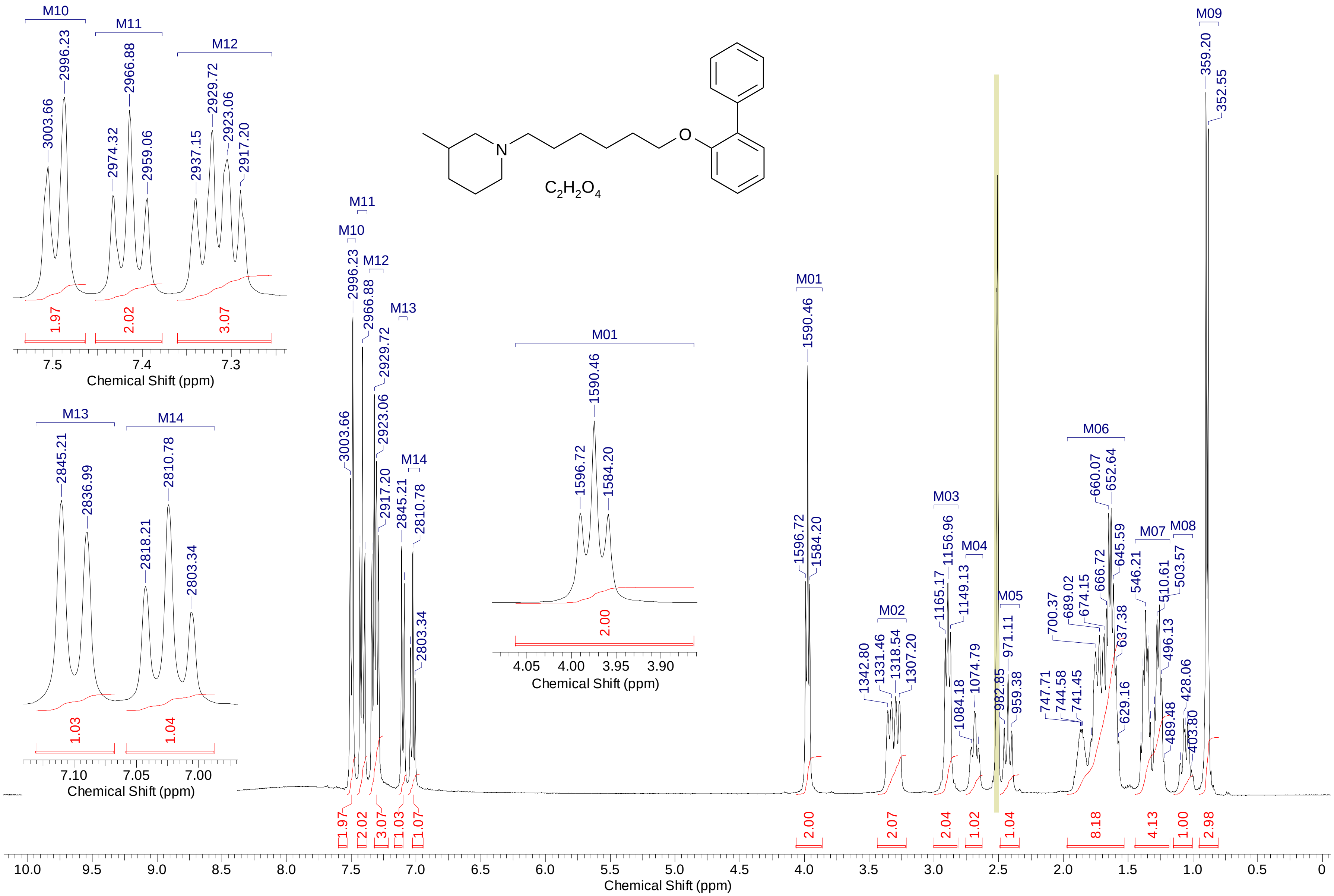

Figure S11. ^{1}H NMR spectrum of 1-(6-([1,1′-biphenyl]-2-yloxy)hexyl)-3-methylpiperidine hydrogen oxalate (**15**).

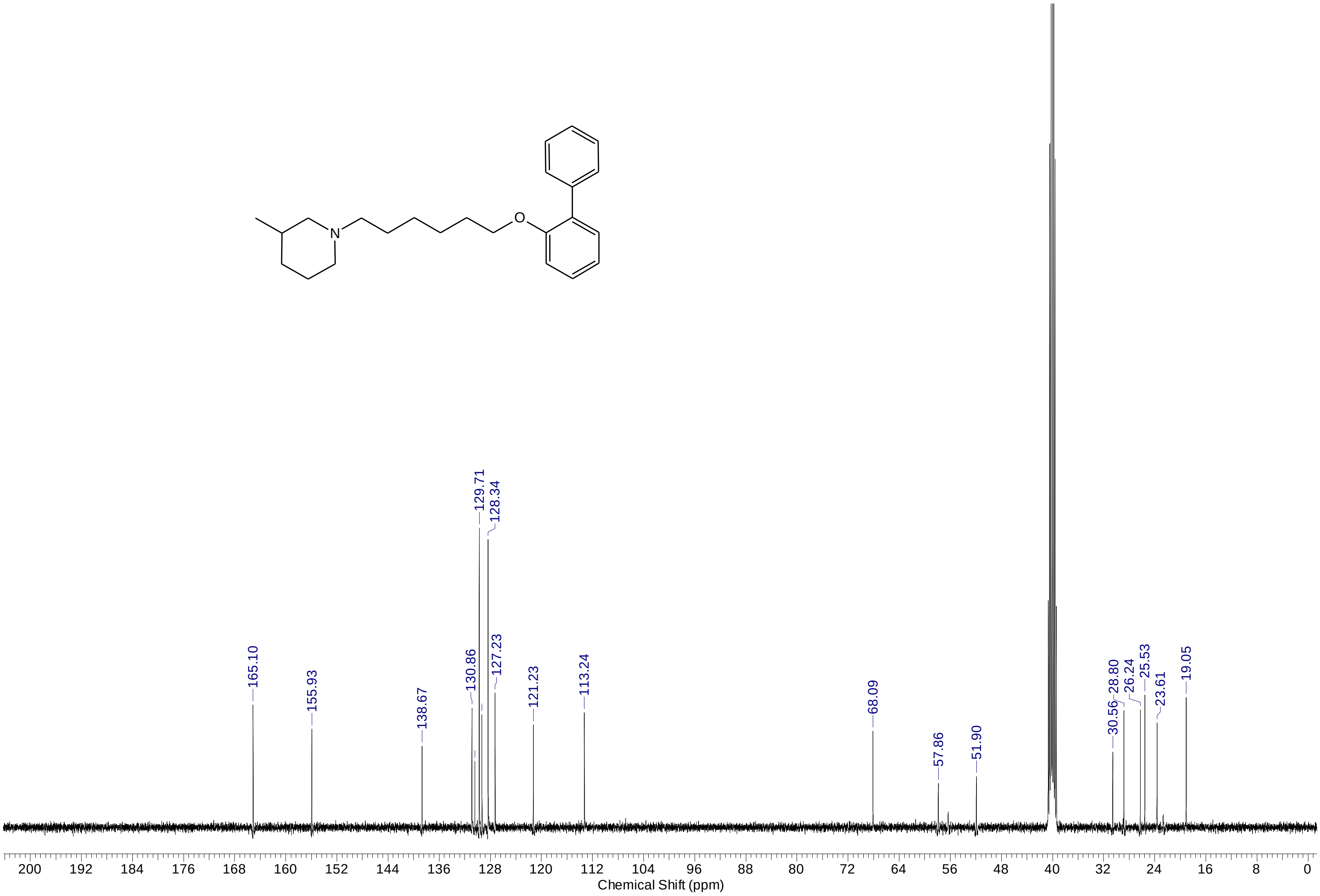

Figure S12. ^{13}C NMR spectrum of 1-(6-([1,1′-biphenyl]-2-yloxy)hexyl)-3-methylpiperidine hydrogen oxalate (**15**).

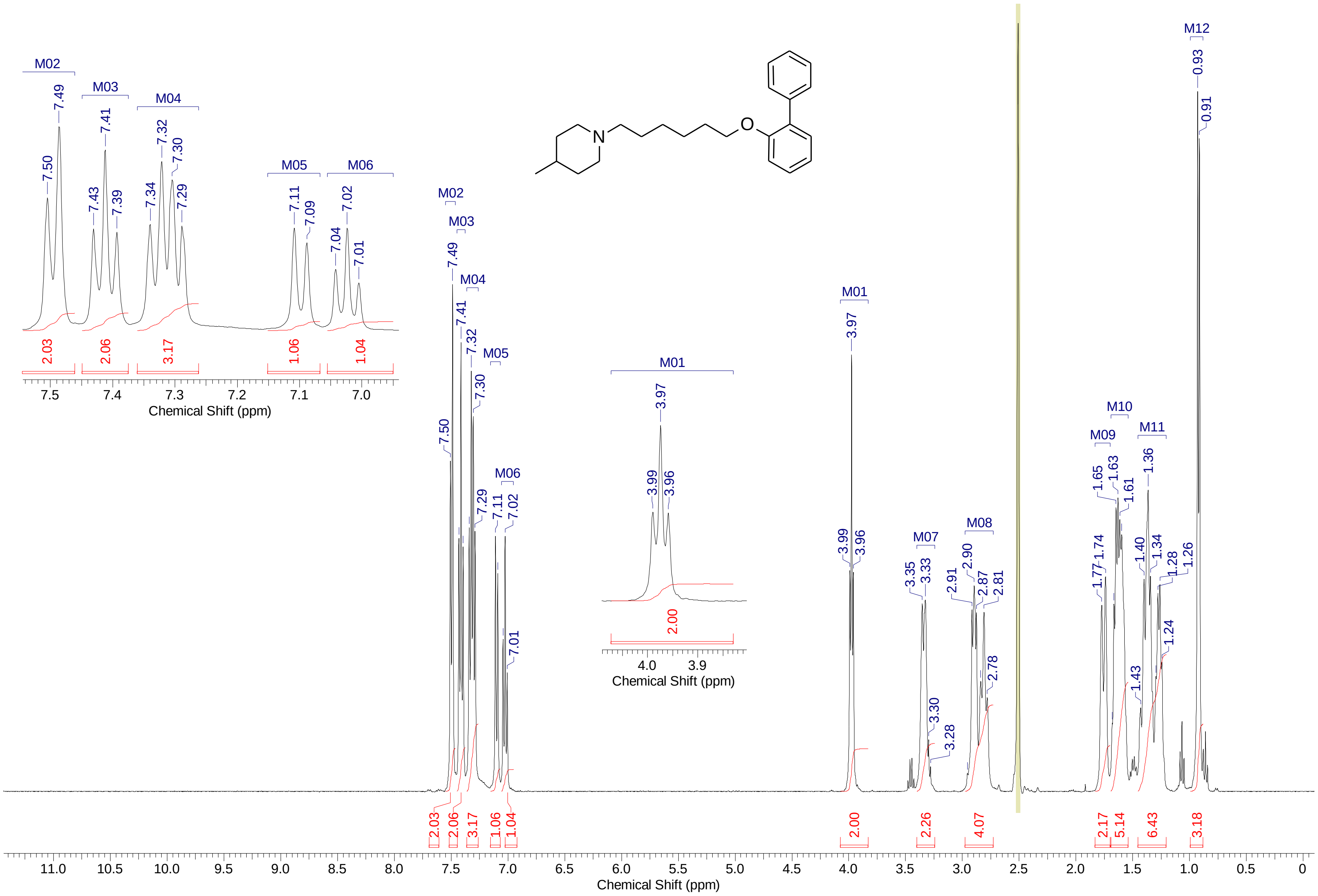

Figure S13. ^{1}H NMR spectrum of 1-(6-([1,1′-biphenyl]-2-yloxy)hexyl)-4-methylpiperidine hydrogen oxalate (**18**).

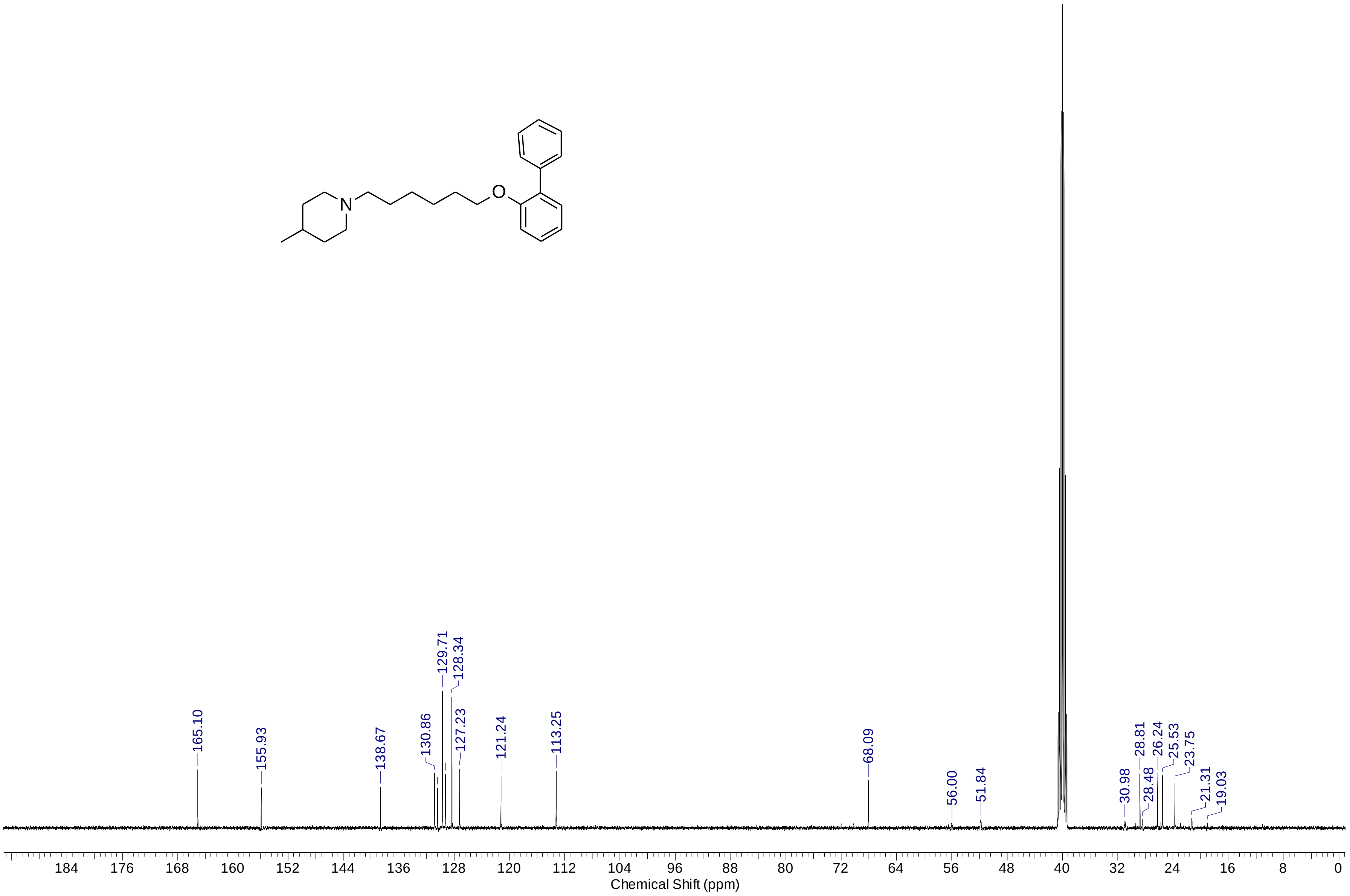

Figure S14. ^{13}C NMR spectrum of 1-(6-([1,1′-biphenyl]-2-yloxy)hexyl)-4-methylpiperidine hydrogen oxalate (**18**).

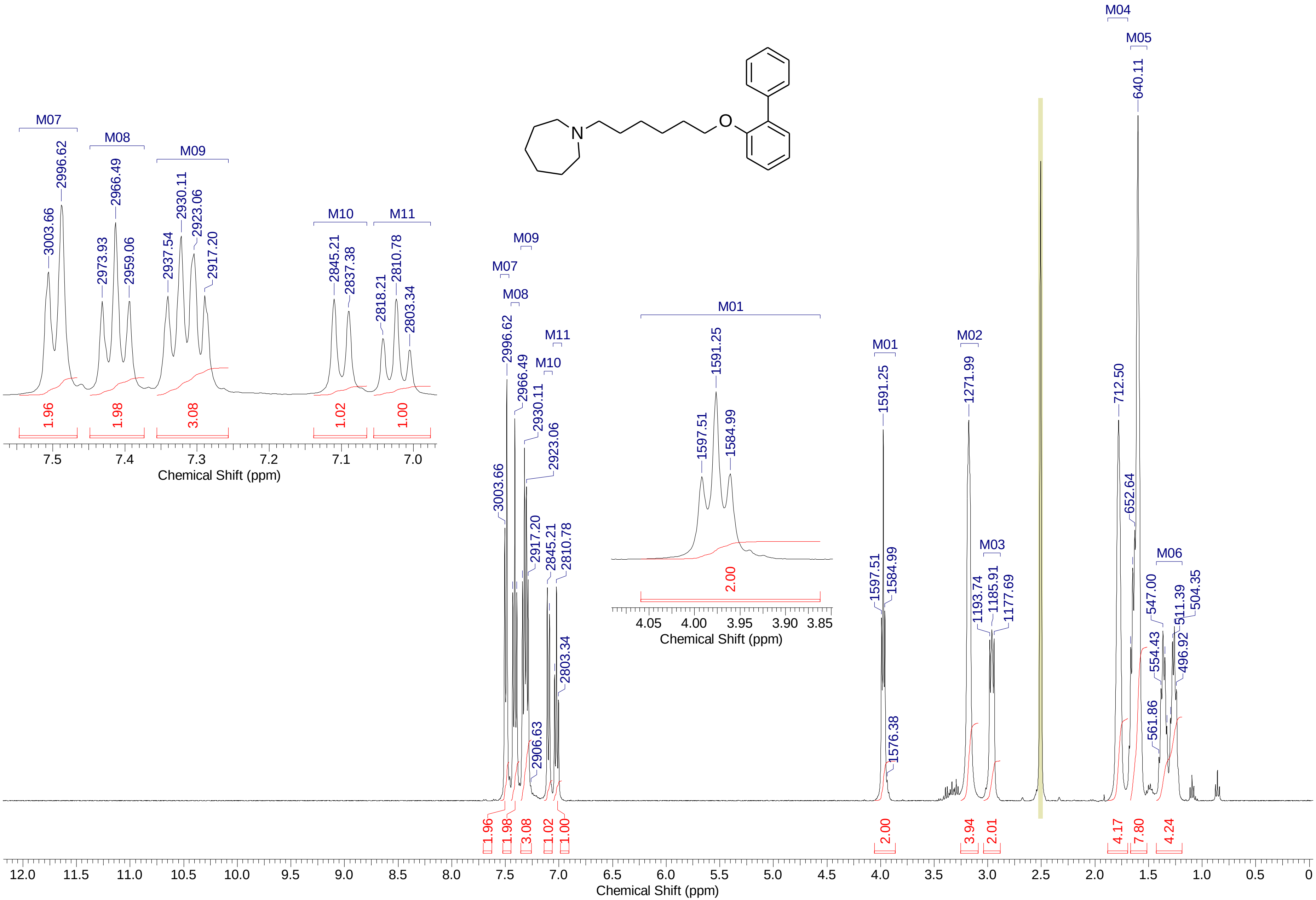

Figure S15. ^{1}H NMR spectrum of 1-(6-([1,1′-biphenyl]-2-yloxy)hexyl)azepane hydrogen oxalate (**22**).

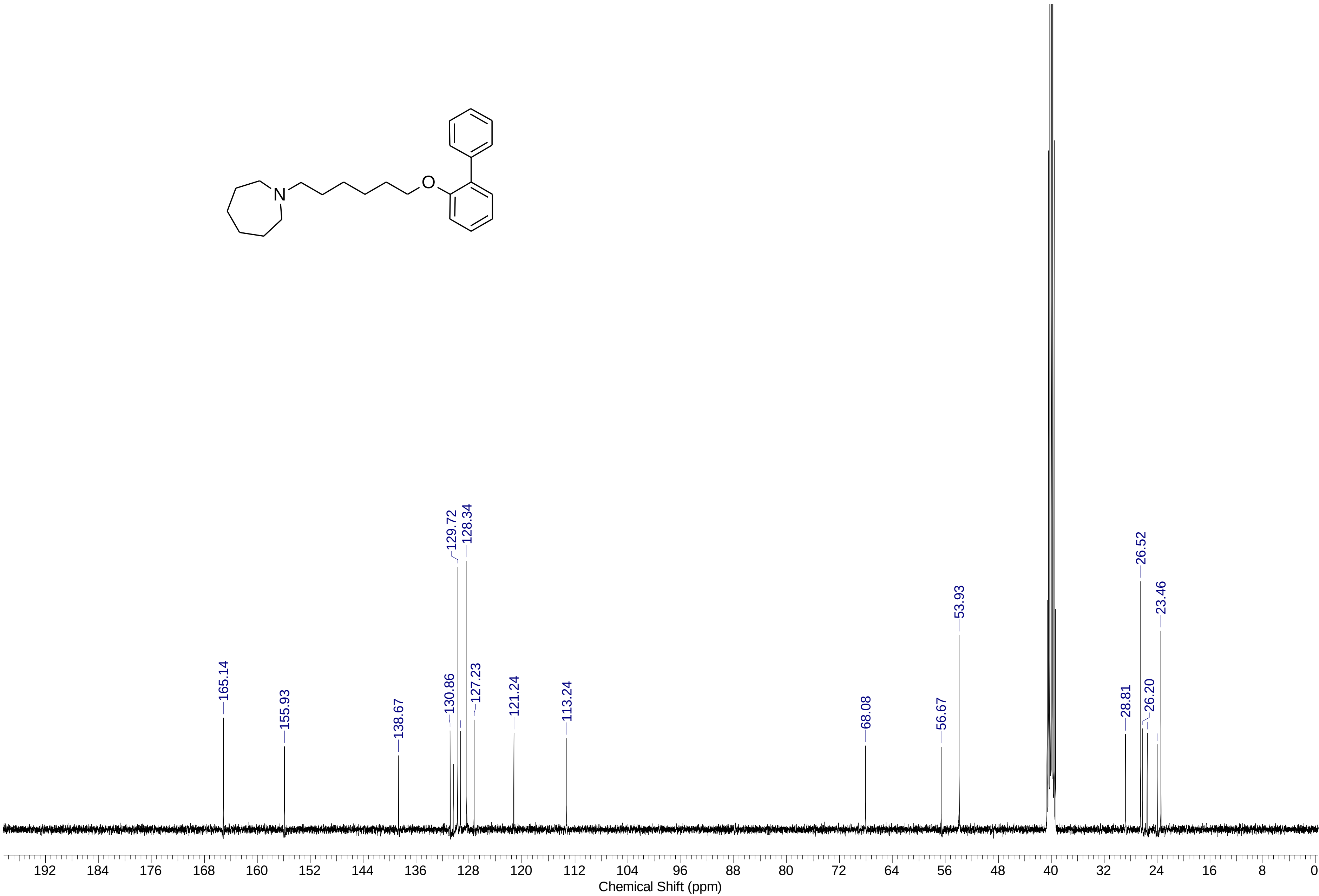

Figure S16. ^{13}C NMR spectrum of 1-(6-([1,1′-biphenyl]-2-yloxy)hexyl)azepane hydrogen oxalate (**22**).
